# Unraveling ethnic disparities in antipsychotic prescribing among patients with psychosis: A retrospective cohort study based on electronic clinical records

**DOI:** 10.1016/j.schres.2023.08.024

**Published:** 2023-10

**Authors:** Tao Wang, David Codling, Dinesh Bhugra, Yamiko Msosa, Matthew Broadbent, Rashmi Patel, Angus Roberts, Philip McGuire, Robert Stewart, Richard Dobson, Robert Harland

**Affiliations:** a Institute of Psychiatry, Psychology and Neuroscience, King's College London, Denmark Hill, London SE5 8AF, United Kingdom; b South London and Maudsley National Health Service (NHS) Foundation Trust, Denmark Hill, London SE5 8AZ, United Kingdom; c Department of Psychiatry, University of Oxford, Oxford, United Kingdom; d Oxford Health, Oxford Health NHS Foundation Trust, Oxford, United Kingdom; e Institute of Health Informatics, University College London, Euston Road, London NW1 2DA, United Kingdom; f Health Data Research UK London, University College London, Euston Road, London NW1 2DA, United Kingdom

**Keywords:** Healthcare inequality, Ethnicity, Psychosis, Antipsychotic prescription, Electronic health records, Psychopharmacology

## Abstract

**Background:**

Previous studies have shown mixed evidence on ethnic disparities in antipsychotic prescribing among patients with psychosis in the UK, partly due to small sample sizes. This study aimed to examine the current state of antipsychotic prescription with respect to patient ethnicity among the entire population known to a large UK mental health trust with non-affective psychosis, adjusting for multiple potential risk factors.

**Methods:**

This retrospective cohort study included all patients (*N* = 19,291) who were aged 18 years or over at their first diagnoses of non-affective psychosis (identified with the ICD-10 codes of F20–F29) recorded in electronic health records (EHRs) at the South London and Maudsley NHS Trust until March 2021. The most recently recorded antipsychotic treatments and patient attributes were extracted from EHRs, including both structured fields and free-text fields processed using natural language processing applications. Multivariable logistic regression models were used to calculate the odds ratios (OR) for antipsychotic prescription according to patient ethnicity, adjusted for multiple potential contributing factors, including demographic (age and gender), clinical (diagnoses, duration of illness, service use and history of cannabis use), socioeconomic factors (level of deprivation and own-group ethnic density in the area of residence) and temporal changes in clinical guidelines (date of prescription).

**Results:**

The cohort consisted of 43.10 % White, 8.31 % Asian, 40.80 % Black, 2.64 % Mixed, and 5.14 % of patients from Other ethnicity. Among them, 92.62 % had recorded antipsychotic receipt, where 24.05 % for depot antipsychotics and 81.72 % for second-generation antipsychotic (SGA) medications. Most ethnic minority groups were not significantly different from White patients in receiving any antipsychotic. Among those receiving antipsychotic prescribing, Black patients were more likely to be prescribed depot (adjusted OR 1.29, 95 % confidence interval (CI) 1.14–1.47), but less likely to receive SGA (adjusted OR 0.85, 95 % CI 0.74–0.97), olanzapine (OR 0.82, 95 % CI 0.73–0.92) and clozapine (adjusted OR 0.71, 95 % CI 0.6–0.85) than White patients. All the ethnic minority groups were less likely to be prescribed olanzapine than the White group.

**Conclusions:**

Black patients with psychosis had a distinct pattern in antipsychotic prescription, with less use of SGA, including olanzapine and clozapine, but more use of depot antipsychotics, even when adjusting for the effects of multiple demographic, clinical and socioeconomic factors. Further research is required to understand the sources of these ethnic disparities and eliminate care inequalities.

## 1 Introduction

An elevated risk in the incidence of psychotic disorders has been consistently reported for several decades among ethnic minority groups compared to the White majority in the UK (Halvorsrud et al., 2018; Halvorsrud et al., 2019; Nazroo et al., 2020), and other countries including the Netherlands (Veling et al., 2007) and the USA (Williams and Mohammed, 2009). The over-representation of people from ethnic minority groups receiving psychosis diagnoses has led to growing concerns over prescribing inequality by ethnicity, which entails ethnic differences in prescribing practices that are not due to access-related factors, clinical needs, preferences, or appropriateness of intervention (Egede, 2006; McCartney et al., 2019), as such inequality may reflect discriminatory policies within a healthcare system and/or discriminatory practices from individual healthcare staff (Das-Munshi et al., 2018; D'Anna et al., 2018).

Prescriptions of antipsychotics, a class of psychotropic medications used to manage psychosis such as olanzapine and clozapine (Kuipers et al., 2014), have been particularly of interest in the literature (Das-Munshi et al., 2018; Taylor, 2004; Connolly and Taylor, 2008; Williams et al., 2020), as most treatment pathways for psychosis involve the prescribing of antipsychotics (Kuipers et al., 2014). A recent systematic review on studies mainly from the USA found that patients from ethnic minority groups had lower prescribing rates of clozapine, a second-generation antipsychotic (SGA) that is widely used in treatment-resistant schizophrenia, when compared with the White patients consistently across time and settings (Williams et al., 2020). In contrast, research from the UK is relatively limited and has either focused on patients under a single type of care services such as an inpatient or outpatient setting (Taylor, 2004; Connolly and Taylor, 2008; Connolly et al., 2007), with a relatively small sample size, or a particular psychotic disorder, such as schizophrenia and schizoaffective disorder, rather than the wide spectrum of psychosis (Das-Munshi et al., 2018). These studies have shown mixed results, where no difference has been found in antipsychotic prescription between Black and White patients (Connolly and Taylor, 2008; Connolly et al., 2007), while Black patients have been shown to be less likely to receive clozapine relative to White groups (Das-Munshi et al., 2018). These mixed results highlight the need for further investigation on the relationship between antipsychotic prescription and ethnicity based on a large population of patients with a wide range of psychotic disorders from both inpatient and outpatient caseloads across mental healthcare services. Moreover, the Covid-19 pandemic has triggered extensive changes in healthcare provision and socioeconomic dynamics (Topriceanu et al., 2021), and has been shown to have widened existing health inequalities, particularly impacting women, ethnic minorities and those with chronic illnesses (Topriceanu et al., 2021; Feinberg et al., 2022). However, no up-to-date studies have examined the current antipsychotic prescribing practices and accessed the impact of the pandemic on ethnic disparities in antipsychotic prescribing.

Research on ethnicity and health indicates that ethnicity can link to health through many possible pathways, including genetic profile, culture, ethnic identity, socioeconomic status and discrimination (Egede, 2006; Ingleby, 2012; Stronks et al., 2013). These explanatory mechanisms can possibly underlie the association between ethnicity and antipsychotic prescribing practice as well. For example, initiation of a long-acting depot antipsychotic is often related to clinicians' perception of non-compliance to medication (Kuipers et al., 2014; Patel and David, 2005; Kim et al., 2020), while non-compliance is dependent upon a number of cultural factors, such as the explanatory models and how cultures of ethnic groups see the use of medication (Bhugra et al., 2015; Li et al., 2012), and health-related behaviours that can vary across ethnic groups such as cannabis smoking and substance use (Stronks et al., 2013; Westermeyer, 1984; Abbot and Chase, 2008). Other studies also found that depots were more likely to be initiated in homeless patients and those who used methods other than private insurance to pay for their care (West et al., 2008), suggesting the role of socioeconomic status in receiving prescribing. This raises the question whether observed ethnic disparities in antipsychotic prescribing are due to differences of health-related behaviours and socioeconomic statuses across ethnic groups. Thus, it is important to take various factors, including clinical, demographic, socioeconomic and behavioural characteristics, into account to clarify the sources of ethnic disparities and inform designing effective interventions to reduce disparities.

Over the past decade, electronic health records (EHRs) have been introduced across most clinical service sectors, which provides a useful resource to examine fine-grained patterns of antipsychotic prescribing for large populations (Connolly et al., 2007; Leckman-Westin et al., 2014; Keating et al., 2021). However, mental health prescription data, as well as related contextual information, are often recorded in free text fields (e.g., clinical notes) rather than structured fields (e.g., drop-down lists) (Kadra et al., 2015). Also, most community prescriptions are dispensed from independent pharmacies and primary care services, and there is no complete structured dataset for prescribing information in the absence of a data linkage across different EHR databases. A common data source documenting these community prescriptions within secondary mental health care is correspondence letters and attachments between healthcare professionals. Traditionally, extracting prescribing information from free-text notes and letters requires manual coding (Taylor, 2004; Su et al., 2014), which is time consuming and not feasible for large-scale data. Recent advances have shown that natural language processing (NLP) techniques provide an effective approach enabling automated extraction of clinical information from large-scale EHR data (Kadra et al., 2015; Cunningham et al., 2013; Perera et al., 2016; Kreimeyer et al., 2017), including medication use (Kadra et al., 2015; Jouffroy et al., 2021). This provides novel opportunities for investigating and monitoring antipsychotic prescribing and any systemic inequality at scale.

In this paper, we make use of a comprehensive EHR-derived data resource from a large mental health service in South London, including extensively derived data using NLP. We aim to examine the relationship between ethnicity and antipsychotic prescribing, accounting for a range of patient factors that may influence such prescribing practices. Sensitivity and mediation analysis using different variants of statistical models were also conducted to better understand the mechanism or pathway that underlies an observed relationship between ethnicity and prescribing disparities.

## 2 Methods

### 2.1 Conceptual framework

**Fig. 1:**
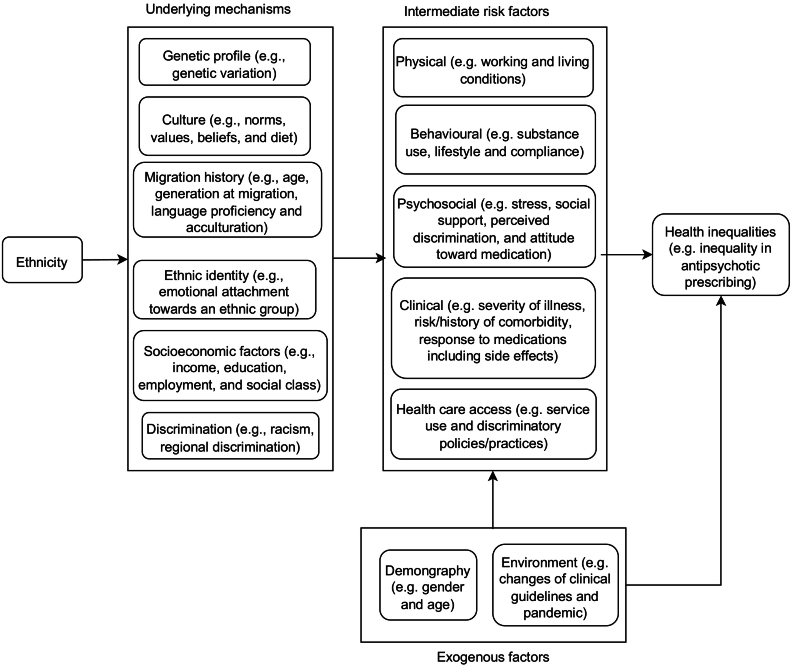
A conceptual framework for the associations between ethnicity and health inequalities, where some variables are grouped into concepts for legibility. Ethnicity can link to health inequalities through underlying mechanisms such as genetic profile, culture, migration history, ethnic identity, socioeconomic factors and discrimination. These mechanisms can be manifested via physical, behavioural, psychosocial, clinical risk factors and health care access, namely intermediate variables between ethnicity and health inequalities in statistical models. Exogenous factors such as demography and environment can influence both a risk factor and health outcome, e.g., age can influence the access to health care and is an important factor in considering a prescription. When examining a causal pathway through a risk factor, these exogenous factors are needed to be adjusted to avoid to confounding effects.

Ethnicity is a complex concept which does not only comprise inherited characteristics such as genetic profile and related physical traits of an ethnic group, but also other aspects that can be learned within the group, such as culture, religion, diet, values and customs (Egede, 2006; Ingleby, 2012; Westermeyer, 1984). In a multicultural society, ethnicity can further comprise a relational dimension, which captures the characteristics of the relationship between an ethnic group and the society that group lives in, such as exposure to discrimination, migration history, ethnic identity, and socioeconomic position (Stronks et al., 2013; Ford and Harawa, 2010). These characteristics can link to health inequalities across ethnic groups through physical, behavioural, psychosocial, clinical risk factors of health, as well as health care access. The relationship between ethnicity and health inequalities can be visualized by a conceptualized version of directed acyclic graph (DAG) (Pearl, 1998) in Fig. 1.

### 2.2 Data source

This study comprised a retrospective examination of recorded treatments for patients with psychosis using EHR data from the South London and Maudsley NHS Foundation Trust (SLaM). SLaM is one of the largest mental health trusts in the UK, providing a wide range of secondary mental health services for a local population of 1.3 million residents in South London and specialist services for people across the UK. Data were extracted using the “Clinical Record Interactive Search” (CRIS), a case register platform which allows authorised researchers to retrieve and analyze anonymized extracts of the entire EHR within a robust information governance framework (Stewart et al., 2009). CRIS combines structured data and information extracted from unstructured text data of clinical notes by NLP algorithms (Perera et al., 2016). Secondary analysis of CRIS data was approved by Oxford Research Ethics Committee C (reference 18/SC/0372) and this study was approved by the CRIS Oversight Committee.

### 2.3 Sample

Participants were included if they were aged 18 years or over at their first diagnoses of schizophrenia, schizotypal or delusional disorders, identified by the International Classification of Diseases, Tenth Revision (ICD-10) codes of F20–F29, recorded as primary or secondary diagnoses at any point up to 26 March 2021 (inclusive). Both active and discharged patients with these diagnoses were included, where information for patients who were no longer active to the Trust was recorded until the date of discharge.

### 2.4 Outcome variables

The primary outcome variable was the “current” or latest antipsychotic prescribed up to the date of data collection for each patient, including whether this was given as an oral or long-acting injectable (depot) medication. Prescription information was extracted from all structured fields (e.g., medication, treatment plan and pharmacy dispensing tables) and free-text data (e.g., event notes recorded in clinician-patient encounters and correspondence letters between healthcare professionals) for recording medication and pharmacy dispensing information in EHR. Prescription information in free text was extracted using a NLP application which extracted mentions of medications in text by using drug name gazetteers and then used rules to exclude irrelevant mentions. The application was developed based on the General Architecture for Text Engineering (GATE) software, a suite of tools used for diverse NLP tasks such as text parser, morphology, tagging and information extraction (Cunningham et al., 2013) and has been regularly validated (with a precision of 96 % and a recall of 90 % reported^1^) and deployed to extract clinical information from routinely collected EHR in CRIS. Development and validation details on the NLP applications deployed in CRIS have been reported in previous publications (Kadra et al., 2015; Cunningham et al., 2013; Perera et al., 2016) and online documents.^2^ Where multiple medications were mentioned in the same document, we prioritized a medication with mentioning a dose in the document, and then prioritized medications considered to be more likely to be prescribed according to clinical guidelines (Kuipers et al., 2014). In an audit of 200 documents, the medication identified as most current was concordant with the clinician 94 % of the time.

To categorize whether an antipsychotic was given as an oral or depot medication, the following method was used. Medications that exist only in depot form were assigned as depot and those that exist only as oral medications were assigned as non-depot. For medications that can be given orally or as depot, an algorithm based on dose, i.e., a mention of a medication with a dose higher than a standard oral dose that is recommended by the British National Formulary (BNF) (Prasad, 1994) was assigned as depot, was devised and validated against a clinician's (DC) view as to whether a mention represented depot or oral medications. In the same audit, the algorithm correctly identified depot vs non-depot with an accuracy of 100 %.

Note that we used the latest antipsychotic as the main outcome for the following reasons. First, the focus of this paper is to examine the current state of antipsychotic prescription among patients with psychosis, which is likely to be reflected by the latest antipsychotic prescription. Second, the majority of prescribing information in mental health records was recorded as free text. However, temporal information about prescribing was often implicitly recorded, e.g., a medication can be prescribed “at present” or “before” or “future (hypothetical)” prescriptions at the time of writing a clinical note. It is still technically challenging and is an active research area to extract reliable temporal information of prescriptions and retrieve the longitudinal data on prescriptions from clinical text (Alfattni et al., 2020). As a trade-off solution, we used handcrafted rules to select the latest prescription from prescriptions extracted by NLP methods and validate the results through clinicians' annotations. Finally, using the latest prescription makes it easier to interpret how different factors (e.g., inpatient bed days and cannabis use) are associated with the prescribing outcome and avoids simultaneity in statistical models, since these factors are likely to be observed before the latest prescription.

### 2.5 Explanatory variable

Our main explanatory variable was patient ethnicity. Given small sizes for some ethnic groups, we classified the source 18 groups from the EHR to 5 categories based on the grouping method used in the 2011 Census of England and Wales: i) Asian (including Bangladeshi, Chinese, Indian, Pakistani, and Asian other), ii) Black (including Black African, Black Caribbean, and Black other), iii) Mixed (including Mixed White/Asian, Mixed White/Black African, Mixed White/Black Caribbean, and Mixed other), iv) White (including White British, White Irish, White Gypsy/Traveller, and White other) and v) Other (including Arab, and Any other).

### 2.6 Covariates

Based on the framework in Fig. 1, we identified a number of covariates for statistical models, including gender, age at the first inclusion diagnosis, illness duration from the date of the first inclusion diagnosis of psychosis until 26 March 2021, and three measures characterizing severity of psychotic illness: i) a categorical variable indicating the latest or current psychotic disorder diagnosis (“F20” — schizophrenia, “F21” — schizotypal disorder, “F22” — persistent delusional disorders, “F23” — acute and transient psychotic disorders, “F24” — induced delusional disorder, “F25” — schizoaffective disorders, “F28” — other nonorganic psychotic disorders or “F29” — unspecified nonorganic psychosis), ii) a binary variable indicating whether a patient was managed by an early intervention (EI) in psychosis team at the time of observation, and iii) intensity of inpatient service use defined as the total number of inpatient episodes and the total number of bed days in SLaM. Note that the covariate on the latest psychotic disorder diagnosis can also control for the effects of ethnic inequalities and any potentially pre-existing racism in diagnosing a specific psychotic disorder on the prescribing inequalities of interest, and these variables on service use can help adjust for variances in prescribing practices across different services.

We also included variables on history of substance use, socioeconomic status and neighbourhood ethnic density, given their associations with prescription of antipsychotics (Bonnot et al., 2017; Foglia et al., 2017; Termorshuizen et al., 2018). Cannabis use, one of the most commonly used illicit substance among those with psychosis (Foglia et al., 2017), was extracted via NLP and recorded as a binary variable representing whether or not there was evidence of cannabis use recorded in EHR. Neighbourhood-level socioeconomic status included two factors: level of deprivation and own-group ethnic density in the area of residence. Level of deprivation was measured by the Index of Multiple Deprivation 2019 (IMD19), the official measure of relative deprivation for small areas in England (Noble et al., 2006), based on the Lower layer Super Output Area (LSOA) of the most recently recorded residence of a patient in EHR. The IMD19 ranking combines seven different domains of deprivation, including Income Deprivation, Employment Deprivation, Education, Skills and Training Deprivation, Health Deprivation and Disability, Crime, Barriers to Housing and Services and Living Environment Deprivation, where a smaller score of IMD19 indicates a higher level of deprivation. To facilitate interpretation, deciles of IMD19 rankings were used, where 1 is the most deprived 10 % of areas and 10 is the least deprived 10 %. Ethnic density for an ethnic group was defined by the percentage of population in a local authority belonging to the ethnic group, which was calculated in the UK's 2011 Census for 18 ethnic groups.^3^ Moreover, the date of latest antipsychotic prescription was also considered to capture the impact of changes in clinical guidelines over time on the choice of antipsychotics (Keating et al., 2021). Details of covariates are listed in Table S1 of Appendix.

### 2.7 Statistical analyses

Descriptive statistics on patient characteristics and general prescribing patterns were reported and compared across ethnic groups, where frequencies and percentages were reported for categorical variables, and means and standard deviations (SDs) for continuous variables. Non-parametric Kruskal-Wallis *H* (Kruskal and Wallis, 1952) and *χ*^2^ tests were used to test differences across groups, with Bonferroni correction (Curtin and Schulz, 1998) of *p* values reported for multiple comparisons. Multivariable logistic regressions were used to assess the association between ethnicity and outcome of prescribing a given antipsychotic medication, adjusted for gender, age, illness duration, proxies for severity, cannabis use, level of deprivation and own-group ethnic density in the area of residence, and date of the latest prescription. All data were pre-processed in Python 3.7.6, regression analyses were conducted in R 3.6.1 (using the built-in “glm” package) and R package mediation 4.5.0 was used in mediation analysis.

## 3 Results

### 3.1 Cohort characteristics

**Fig. 2:**
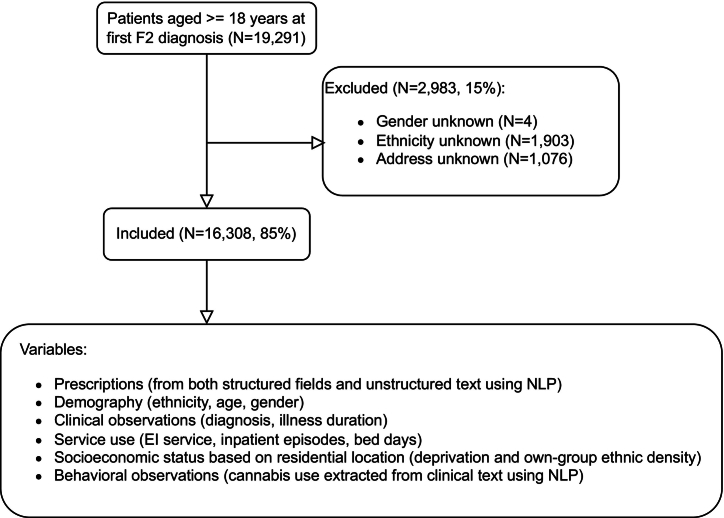
Sample and data flow in the study.

As shown in Fig. 2, we identified 19,291 patients who were aged at least 18 years at first F20–F29 diagnosis until 26 March 2021, where the dates of patients' first F2 diagnoses range from 1958 to 2021 while most diagnoses (99.3 %, *N* = 16,200) were recorded since 2000 (see Fig. S1 in Appendix for details). After excluding those who had missing values on gender (*N* = 4), ethnicity (*N* = 1903) and those with invalid addresses (*N* = 1076), the final cohort contained 16,308 individuals.

Table 1 describes the characteristics of the cohort by ethnicity. White patients were older and had a shorter illness duration than other ethnic groups. The majority of the cohort was male (58.35 %), although this disparity was least evident in the Asian population (54.72 %). There were similar distributions of diagnoses across ethnic groups, except patients from the Other group. A higher proportion of ethnic minority patients were managed by an EI team at present. Patients from Black and Mixed groups had more episodes of inpatient treatment (mean 2.1 against 1.5 overall) and were more likely to have evidence of cannabis use recorded than other groups. Compared to the White group, patients from ethnic minority groups tended to live in more deprived areas. Among ethnic minority groups, patients in the Black group had a higher level of own-group ethnic density. Most patients in the cohort had antipsychotic use recorded in recent years, where the dates of patients' latest antipsychotic prescribing range from May 9, 2000 to March 5, 2021 and 53.6 % of all patients who received an antipsychotic prescription had their latest prescriptions recorded after 2018, implying that our data were likely to capture up-to-date practices in prescribing antipsychotics.

**Table 1:** Patient characteristics by ethnicity.

	Full sample		White		Asian		Black		Mixed		Other		*H*/*χ*^2^
	N	%	N	%	N	%	N	%	N	%	N	%	*p*^b^
Total	16,308		7029	43.10 %	1356	8.31 %	6654	40.80 %	431	2.64 %	838	5.13 %	
Age in years^a^	41.9	(16.65)	45.7	(17.78)	41.0	(15.78)	39.1	(15.22)	35.3	(13.83)	38.3	(13.53)	*H* = 599.13 *p* *<* 0.001
Illness duration^a^ (in years)	9.6	(5.85)	9.1	(5.78)	9.5	(5.56)	10.5	(5.94)	9.7	(5.89)	7.6	(4.86)	*H* = 329.03 *p* *<* 0.001
Gender													
Female	6793	41.65 %	2840	40.40 %	614	45.28 %	2840	42.68 %	171	39.68 %	328	39.14 %	*χ*^2^ = 17.62 *p* = 0.016
Male	9515	58.35 %	4189	59.60 %	742	54.72 %	3814	57.32 %	260	60.32 %	510	60.86 %	
Diagnosis													
F20	9416	57.74 %	4073	57.95 %	778	57.37 %	3967	59.62 %	242	56.15 %	356	42.48 %	*χ*^2^ = 248.75 *p* *<* 0.001
F21	102	0.63 %	63	0.90 %	7	0.52 %	24	0.36 %	0	0.00 %	8	0.95 %	
F22	1040	6.38 %	527	7.50 %	93	6.86 %	317	4.76 %	21	4.87 %	82	9.79 %	
F23	1263	7.74 %	498	7.08 %	133	9.81 %	493	7.41 %	26	6.03 %	113	13.48 %	
F24	5	0.03 %	2	0.03 %	0	0.00 %	1	0.02 %	0	0.00 %	2	0.24 %	
F25	2187	13.41 %	988	14.06 %	152	11.21 %	883	13.27 %	62	14.39 %	102	12.17 %	
F28	270	1.66 %	130	1.85 %	17	1.25 %	96	1.44 %	6	1.39 %	21	2.51 %	
F29	2025	12.42 %	748	10.64 %	176	12.98 %	873	13.12 %	74	17.17 %	154	18.38 %	
EI service													
No	14,755	90.48 %	6511	92.63 %	1215	89.60 %	5950	89.42 %	364	84.45 %	715	85.32 %	*χ*^2^ = 91.66 *p* *<* 0.001
Yes	1553	9.52 %	518	7.37 %	141	10.40 %	704	10.58 %	67	15.55 %	123	14.68 %	
#Inpatient episodes^a^	1.5	(2.61)	1.1	(2.22)	1.2	(2.16)	2.1	(2.98)	2.1	(3.31)	1.1	(1.90)	*H* = 776.12 *p* *<* 0.001
#Bed days^a^	169.6	(453.45)	133.8	(407.81)	112.6	(306.65)	227.2	(527.18)	222.4	(514.94)	77.6	(242.66)	*H* = 643.79 *p* *<* 0.001
Cannabis use													
No	7234	44.36 %	3495	49.72 %	779	57.45 %	2439	36.65 %	125	29.00 %	396	47.26 %	*χ*^2^ = 380.11 *p* *<* 0.001
Yes	9074	55.64 %	3534	50.28 %	577	42.55 %	4215	63.35 %	306	71.00 %	442	52.74 %	
IMD19 decile^a^	3.8	(1.89)	4.1	(2.10)	3.9	(1.87)	3.5	(1.57)	3.9	(1.93)	3.8	(1.79)	*H* = 285.00 *p* *<* 0.001
Ethnic density^a^	21.6	(23.35)	41.4	(23.44)	3.7	(3.13)	8.1	(4.29)	1.9	(0.74)	2.0	(0.76)	*H* = 9917.72 *p* *<* 0.001
Latest prescription date^c^													
[2000, 2010)	2000	13.24 %	1066	16.75 %	157	12.29 %	683	10.81 %	35	8.58 %	59	8.04 %	*χ*^2^ = 393.02 *p* *<* 0.001
[2010, 2020)	6902	45.69 %	3160	49.64 %	644	50.43 %	2540	40.19 %	163	39.95 %	395	53.81 %	
[2020, ∞)	6203	41.07 %	2140	33.62 %	476	37.27 %	3097	49.00 %	210	51.47 %	280	38.15 %	

a Means and SDs (in parentheses) for continuous variables.

b *p*-Values were adjusted for multiple comparisons with Bonferroni corrections *p* = *p*′ ∗ *n*, where *n* = 11 is the number of comparisons and *p*′ is the raw *p*-value from a test.

c Only patients receiving an antipsychotic prescription were considered in calculating the percentages of patients whose latest antipsychotic prescriptions were recorded at a given year.

**Fig. 3:**
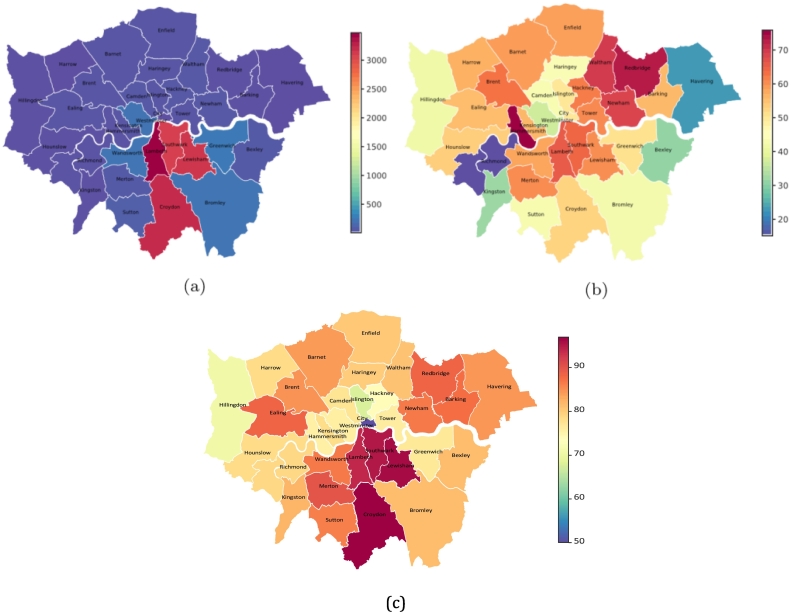
Maps of recorded residences for SLaM patients with psychosis (on 26 March 2021) within London boroughs. (a) Numbers of SLaM patients with psychosis in each London borough. (b) Proportions of patients from minority ethnic groups in London boroughs. (c) Proportions of patients recorded antipsychotic use in London boroughs.

Of the whole cohort, 92.3 % (*N* = 15,050) were residing in London areas and Fig. 3a visualizes the geographic distribution of these patients within London boroughs. The highest numbers of patients with psychosis were found in the four boroughs of SLaM catchment: Lambeth (*N* = 3468, 21.2 %), Croydon (*N* = 3235, 19.8 %), Lewisham (*N* = 3158, 19.4 %) and Southwark (*N* = 3112, 19.1 %). Of the catchment areas, Lambeth (65.9 %) had the highest proportion of patients from minority ethnic groups (Asian, Black, Mixed or Other), followed by Southwark (64.5 %), Lewisham (60.4 %) and Croydon (52.8 %) (Fig. 3b).

### 3.2 Prescribing characteristics

Table 2 displays proportions of recorded antipsychotic use outcomes by ethnicity. Most patients (92.62 %) had received an antipsychotic medication ever recorded, although the rates were lower in the White and Other groups. Higher proportions of patients with antipsychotic use were found in the SLaM catchment boroughs than other areas (Fig. 3c). Among those receiving antipsychotics, Black patients were more likely to be prescribed depot agents. Second-generation antipsychotic (SGA) medications were prescribed more commonly than first-generation antipsychotics (FGAs) across all ethnic groups. Among SGA medications, olanzapine, risperidone, aripiprazole and clozapine were the most commonly prescribed, while flupentixol, zuclopenthixol and haloperidol were the most common FGA medications. Comparing prescribing rates in each ethnic group, most medications had similar distributions across groups, except clozapine which was the third most common antipsychotic in the White group and was the fourth most common antipsychotic across ethnic minority groups.

**Table 2:** Antipsychotic prescriptions by ethnicity.

	Full sample		White		Asian		Black		Mixed		Other		*χ*^2^
	N	%	N	%	N	%	N	%	N	%	N	%	*p*^b^
Total	16,308		7029	43.10 %	1356	8.31 %	6654	40.80 %	431	2.64 %	838	5.14 %	
Prescribed an antipsychotic													
No	1203	7.38 %	663	9.43 %	79	5.83 %	334	5.02 %	23	5.34 %	104	12.41 %	*χ*^2^ = 136.06 *p* *<* 0.001
Yes	15,105	92.62 %	6366	90.57 %	1277	94.17 %	6320	94.98 %	408	94.66 %	734	87.59 %	
Prescribed depot vs oral^a^													
Depot	3632	24.05 %	1367	21.47 %	243	19.03 %	1800	28.48 %	90	22.06 %	132	17.98 %	*χ*^2^ = 124.39 *p* *<* 0.001
Oral	11,473	75.95 %	4999	78.53 %	1034	80.97 %	4520	71.52 %	318	77.94 %	602	82.02 %	
Prescribed SGA vs FGA^a^													
SGA	12,344	81.72 %	5155	80.98 %	1090	85.36 %	5113	80.90 %	340	83.33 %	646	88.01 %	*χ*^2^ = 36.65 *p* *<* 0.001
FGA	2761	18.28 %	1211	19.02 %	187	14.64 %	1207	19.10 %	68	16.67 %	88	11.99 %	
Antipsychotic prescribed^a^													*χ*^2^ = 487.89 *p* *<* 0.001
SGA													
Amisulpride	685	4.53 %	317	4.98 %	57	4.46 %	261	4.13 %	17	4.17 %	33	4.50 %	
Aripiprazole	2144	14.19 %	741	11.64 %	210	16.44 %	1009	15.97 %	62	15.20 %	122	16.62 %	
Cariprazine	19	0.13 %	4	0.06 %	3	0.23 %	10	0.16 %	0	0.00 %	2	0.27 %	
Clozapine	1698	11.24 %	890	13.98 %	129	10.10 %	562	8.89 %	60	14.71 %	57	7.77 %	
Lurasidone	111	0.73 %	39	0.61 %	7	0.55 %	55	0.87 %	6	1.47 %	4	0.54 %	
Olanzapine	3681	24.37 %	1602	25.16 %	320	25.06 %	1476	23.35 %	79	19.36 %	204	27.79 %	
Paliperidone	924	6.12 %	260	4.08 %	68	5.32 %	533	8.43 %	28	6.86 %	35	4.77 %	
Quetiapine	748	4.95 %	367	5.77 %	72	5.64 %	242	3.83 %	18	4.41 %	49	6.68 %	
Risperidone	2331	15.43 %	934	14.67 %	224	17.54 %	964	15.25 %	70	17.16 %	139	18.94 %	
Ziprasidone hydrochloride^c^	3	0.02 %	1	0.02 %	0	0.00 %	1	0.02 %	0	0.00 %	1	0.14 %	
FGA													
Chlorpromazine	57	0.38 %	34	0.53 %	3	0.23 %	20	0.32 %	0	0.00 %	0	0.00 %	
Droperidol	2	0.01 %	1	0.02 %	1	0.08 %	0	0.00 %	0	0.00 %	0	0.00 %	
Flupentixol	836	5.53 %	390	6.13 %	60	4.70 %	348	5.51 %	19	4.66 %	19	2.59 %	
Fluphenazine	169	1.12 %	99	1.56 %	12	0.94 %	52	0.82 %	2	0.49 %	4	0.54 %	
Haloperidol	607	4.02 %	223	3.50 %	41	3.21 %	299	4.73 %	18	4.41 %	26	3.54 %	
Melleril	1	0.01 %	1	0.02 %	0	0.00 %	0	0.00 %	0	0.00 %	0	0.00 %	
Melperone	3	0.02 %	2	0.03 %	1	0.08 %	0	0.00 %	0	0.00 %	0	0.00 %	
Penfluridol	11	0.07 %	0	0.00 %	2	0.16 %	8	0.13 %	0	0.00 %	1	0.14 %	
Pericyazine	1	0.01 %	0	0.00 %	0	0.00 %	0	0.00 %	0	0.00 %	1	0.14 %	
Perphenazine	1	0.01 %	0	0.00 %	0	0.00 %	0	0.00 %	0	0.00 %	1	0.14 %	
Pimozide	8	0.05 %	3	0.05 %	0	0.00 %	4	0.06 %	1	0.25 %	0	0.00 %	
Piportil	82	0.54 %	38	0.60 %	3	0.23 %	38	0.60 %	2	0.49 %	1	0.14 %	
Pipotiazine	44	0.29 %	13	0.20 %	4	0.31 %	24	0.38 %	1	0.25 %	2	0.27 %	
Promazine	4	0.03 %	3	0.05 %	0	0.00 %	1	0.02 %	0	0.00 %	0	0.00 %	
Sulpiride	143	0.95 %	80	1.26 %	8	0.63 %	48	0.76 %	3	0.74 %	4	0.54 %	
Trifluoperazine	102	0.68 %	53	0.83 %	10	0.78 %	35	0.55 %	0	0.00 %	4	0.54 %	
Zuclopenthixol	690	4.57 %	271	4.26 %	42	3.29 %	330	5.22 %	22	5.39 %	25	3.41 %	

a Only patients who received an antipsychotic prescription were considered in calculating the percentages of patients under depot or oral, second-generation antipsychotic (SGA) or first-generation antipsychotic (FGA) and individual antipsychotics.

b *p*-Values were adjusted for multiple comparisons with Bonferroni corrections *p* = *p*′ ∗ *n*, where *n* = 4 is the number of comparisons and *p*′ is the raw *p*-value from a test.

c Unlicensed medication in the UK but recorded as being used by patients, particularly for those from abroad.

### 3.3 Antipsychotic prescribing and ethnicity

Table 3 displays adjusted odds ratios for the associations between ethnicity and antipsychotic prescription indicators estimated from multivariable logistic regression models. Specifically, Model 1 estimated the associations between patient characteristics and recording of any antipsychotic use. The results of Model 1 show that, after accounting for all other factors, most ethnic minority groups were not significantly different to the White group in receiving an antipsychotic treatment, except the Other group who were less likely to have antipsychotic use recorded. On analyzing each specific type of antipsychotics, Black patients were more likely to be receiving depot antipsychotics (Model 2) and less likely to be receiving SGA medications than the White group (Model 3). However, other ethnic minority groups were not significantly different from the White group with respect to depot and SGA antipsychotic receipt. Inspecting individual antipsychotics, all ethnic minority groups were less likely to receive olanzapine than the White group (Model 4). In contrast, mixed results were observed in associations between ethnicity and clozapine receipt which was less likely in Black patients and more likely in other ethnic minority groups, compared to the White group (Model 5).

**Table 3:** Odds ratios of logistic regressions correlating patient characteristics and antipsychotic prescriptions.^d^

	Dependent variable				
	Prescribed an antipsychotic	Prescribed depot^b^	Prescribed SGA^b^	Prescribed olanzapine^b^	Prescribed clozapine^b^
	(1)	(2)	(3)	(4)	(5)
Ethnicity (ref = “White”) Asian	1.23 (0.92, 1.66)	0.94 (0.78, 1.14)	1.09 (0.89, 1.34)	0.78^∗∗^ (0.66, 0.92)	1.31^∗^ (1.02, 1.69)
Black	0.90 (0.74, 1.10)	1.29^∗∗∗^ (1.14, 1.47)	0.85^∗^ (0.74, 0.97)	0.82^∗∗∗^ (0.73, 0.92)	0.71^∗∗∗^ (0.60, 0.85)
Mixed	0.85 (0.53, 1.40)	0.93 (0.70, 1.22)	0.87 (0.65, 1.18)	0.60^∗∗∗^ (0.46, 0.79)	1.42^∗^ (1.00, 1.99)
Other	0.63^∗∗^ (0.47, 0.84)	1.02 (0.81, 1.29)	1.05 (0.81, 1.37)	0.82^∗^ (0.67, 0.99)	1.12 (0.79, 1.55)
Age	1.02^∗∗∗^ (1.01, 1.02)	1.01^∗∗∗^ (1.01, 1.01)	0.98^∗∗∗^ (0.97, 0.98)	0.99^∗∗∗^ (0.99, 1.00)	0.96^∗∗∗^ (0.96, 0.97)
Illness duration	1.07^∗∗∗^ (1.06, 1.09)	1.00 (0.99, 1.01)	0.98^∗∗∗^ (0.97, 0.99)	1.00 (0.99, 1.01)	1.02^∗∗∗^ (1.01, 1.03)
Gender (ref = “Male”) Female	1.16^∗^ (1.02, 1.33)	0.87^∗∗^ (0.80, 0.95)	1.06 (0.97, 1.16)	0.84^∗∗∗^ (0.78, 0.92)	0.98 (0.87, 1.11)
Diagnosis (ref = “F29”) F20	1.86^∗∗∗^ (1.51, 2.29)	2.88^∗∗∗^ (2.42, 3.44)	0.46^∗∗∗^ (0.38, 0.57)	0.58^∗∗∗^ (0.51, 0.67)	7.98^∗∗∗^ (5.55, 11.89)
F22	0.45^∗∗∗^ (0.35, 0.58)	0.77 (0.57, 1.03)	1.62^∗∗^ (1.19, 2.23)	0.59^∗∗∗^ (0.48, 0.73)	1.28 (0.60, 2.54)
F23	0.54^∗∗∗^ (0.43, 0.69)	0.43^∗∗∗^ (0.31, 0.60)	1.38^∗^ (1.01, 1.89)	1.29^∗∗^ (1.10, 1.52)	0.31^∗^ (0.11, 0.74)
F25	2.69^∗∗∗^ (1.97, 3.71)	2.41^∗∗∗^ (1.98, 2.94)	0.45^∗∗∗^ (0.36, 0.56)	0.70^∗∗∗^ (0.60, 0.82)	5.96^∗∗∗^ (4.07, 9.01)
Other^a^	0.72 (0.52, 1.02)	0.67 (0.41, 1.04)	1.27 (0.82, 2.05)	0.96 (0.74, 1.24)	0.89 (0.30, 2.15)
EI service (ref = “No”) Yes	2.45^∗∗∗^ (1.88, 3.22)	0.73^∗∗^ (0.60, 0.89)	2.20^∗∗∗^ (1.67, 2.94)	0.93 (0.81, 1.07)	0.22^∗∗∗^ (0.13, 0.35)
#Inpatient episodes	6.02^∗∗∗^ (4.95, 7.41)	1.14^∗∗∗^ (1.12, 1.16)	0.94^∗∗∗^ (0.92, 0.95)	0.95^∗∗∗^ (0.93, 0.97)	0.93^∗∗∗^ (0.91, 0.95)
#Bed days	1.00 (1.00, 1.00)	1.00^∗∗∗^ (1.00, 1.00)	1.00 (1.00, 1.00)	1.00 (1.00, 1.00)	1.00^∗∗∗^ (1.00, 1.00)
Cannabis use (ref = “No”) Yes	2.20^∗∗∗^ (1.89, 2.57)	1.19^∗∗∗^ (1.08, 1.31)	1.02 (0.92, 1.13)	0.95 (0.86, 1.04)	1.21^∗∗^ (1.05, 1.39)
IMD19 decile	1.00 (0.97, 1.03)	0.98 (0.96, 1.00)	1.00 (0.98, 1.03)	1.00 (0.98, 1.02)	1.07^∗∗∗^ (1.04, 1.10)
Ethnic density	0.99^∗∗∗^ (0.99, 0.99)	1.00 (1.00, 1.00)	1.00 (0.99, 1.00)	0.99^∗∗∗^ (0.99, 1.00)	1.02^∗∗∗^ (1.01, 1.02)
Date of prescription (ref = “[2000, 2010)”)^c^					
[2010, 2020)		1.04 (0.91, 1.19)	1.47^∗∗∗^ (1.30, 1.67)	0.90 (0.80, 1.00)	1.19 (0.97, 1.48)
[2020, ∞)		1.74^∗∗∗^ (1.51, 2.01)	1.37^∗∗∗^ (1.19, 1.57)	0.57^∗∗∗^ (0.50, 0.65)	2.90^∗∗∗^ (2.35, 3.61)
Constant	1.20 (0.83, 1.74)	0.05^∗∗∗^ (0.04, 0.07)	22.16^∗∗∗^ (16.01, 30.82)	1.20 (0.93, 1.55)	0.02^∗∗∗^ (0.01, 0.03)
Observations	16,308	15,105	15,105	15,105	15,105

a To avoid biased estimates due to small sample issues (Nemes et al., 2009), small-sized diagnoses F21, F24 and F28 were combined together as “Other”.

b Patients without receiving an antipsychotic prescription were excluded in Models 2–5.

c Date of prescription was excluded in Model 1, since samples without an antipsychotic prescription had no information on this predictor by definition.

d Significance of an individual predictor was assessed by the Wald test, where *p *<* 0.05; **p *<* 0.01; and ***p *<* 0.001.

### 3.4 Antipsychotic prescribing and other factors

Compared to male patients, females were more likely to receive antipsychotic treatment but less likely to be recorded as receiving depot agents or olanzapine. Evidence of cannabis use was associated with increased likelihood of overall antipsychotic, depot and clozapine receipt. Living in a less deprived area was associated with a significantly higher likelihood of receiving clozapine. Moreover, a higher own-group ethnic density in the neighbourhood of residence was associated with a lower likelihood of any antipsychotic but a higher likelihood of clozapine receipt. Relative to earlier years prior to 2010, SGA medications were more likely to be recorded in recent years, but olanzapine less likely.

### 3.5 Sensitivity and mediation analyses

Sensitivity analyses were conducted on the relationships between explanatory variables and antipsychotic prescribing outcomes in the regression models (see Appendix). We found that associations between ethnicity and receipt of any antipsychotic showed different patterns before and after adjusting for neighbourhood ethnic density in regression models, suggesting the presence of this variable as a mediator (see mediation analyses in Appendix). Similar results were also observed in estimating the associations between ethnicity and receipt of olanzapine or clozapine. However, the effects of neighbourhood ethnic density on antipsychotic prescribing can vary across ethnic groups. For example, a higher own-group ethnic density in neighbourhood was associated with decreased antipsychotic use in the White and Other groups, while a positive association between ethnic density and antipsychotic use was found in the Black group (Fig. S2, Appendix). Also, cannabis use was consistently observed to show strong associations with the patterns of prescribing any antipsychotic, depot agents and clozapine across different model settings.

### 3.6 Impact of the Covid-19 pandemic

We further compared the associations between patient ethnicity and antipsychotic prescribing estimated based on the prescriptions recorded before and after Jan 1, 2020 respectively to examine the impact of the Covid-19 pandemic on prescribing disparities. As shown in Table 4, compared to the pre-Covid period, ethnic minority groups, particularly the Black patients, were generally more likely than White patients to be prescribed a depot agent, and less likely to receive a SGA and clozapine prescribing during the pandemic. In contrast, inequalities in receiving olanzapine prescribing between ethnic minority groups and the White group became less significant after entering the Covid pandemic.

**Table 4:** Odds ratios of logistic regressions correlating patient ethnicity and antipsychotic prescriptions prior and during the Covid-19 pandemic.^a^

		Dependent variable			
		Prescribed depot^b^	Prescribed SGA^b^	Prescribed olanzapine^b^	Prescribed clozapine^b^
	Ethnicity (ref = “White”) Asian	0.77^∗^ (0.59, 0.99)	1.31^∗^ (1.02, 1.69)	0.77^∗∗^ (0.63, 0.94)	1.48 (0.98, 2.20)
Pre-Covid (*N* = 8902)	Black	1.09 (0.92, 1.29)	1.02 (0.87, 1.21)	0.78^∗∗∗^ (0.68, 0.89)	0.95 (0.71, 1.27)
	Mixed	0.77 (0.49, 1.18)	1.42 (0.92, 2.30)	0.50^∗∗∗^ (0.34, 0.71)	2.55^∗∗∗^ (1.44, 4.35)
	Other	0.90 (0.64, 1.24)	1.45^∗^ (1.03, 2.07)	0.68^∗∗^ (0.53, 0.87)	1.10 (0.61, 1.89)
					
	Ethnicity (ref = “White”) Asian	1.18 (0.89, 1.58)	0.78 (0.55, 1.12)	0.78 (0.56, 1.07)	0.98 (0.69, 1.38)
Covid (*N* = 6203)	Black	1.53^∗∗∗^ (1.26, 1.87)	0.61^∗∗∗^ (0.48, 0.78)	0.90 (0.73, 1.12)	0.50^∗∗∗^ (0.40, 0.64)
	Mixed	1.14 (0.78, 1.66)	0.50^∗∗^ (0.32, 0.78)	0.80 (0.51, 1.21)	0.80 (0.50, 1.24)
	Other	1.20 (0.85, 1.70)	0.65^∗^ (0.42, 1.00)	1.17 (0.82, 1.66)	0.90 (0.58, 1.38)

a Significance of an individual predictor was assessed by the Wald test, where ^∗^*p* *<* 0.05; ^∗∗^*p* *<* 0.01; and ^∗∗∗^*p* *<* 0.001.

b Patients without receiving an antipsychotic prescription were excluded in Models 2–5.

## 4 Discussion

### 4.1 Summary of findings

This study investigated disparities in antipsychotic prescribing by ethnicity drawing on a large and representative population with non-affective psychosis in a highly diverse urban catchment area. Most people with psychosis from ethnic minority groups did not significantly differ from their White counterparts in receipt of an antipsychotic medication. Black patients were more likely than White patients to be prescribed a depot agent, and less likely to receive a SGA, olanzapine or clozapine specifically. All ethnic minority groups were less likely to be prescribed olanzapine than the White group. These associations persisted after adjustment for multiple covariates, including demographic attributes (age and gender), clinical factors (diagnoses, duration of illness, history of cannabis use), service use (EI and inpatient services), socioeconomic status (level of deprivation and own-group ethnic density in the area of residence), and temporal changes in clinical guidelines (date of prescription recorded). Our results also suggested that the Covid-19 pandemic was likely to exacerbate the ethnic disparities in prescribing a depot agent, SGA or clozapine.

### 4.2 Comparison with previous research

Our cohort represents the entire population with non-affective psychosis known to a large mental healthcare provider serving a specified geographic catchment area in South London. It thus comprises a large group of patients with diverse diagnoses receiving input from a variety of mental healthcare teams and is likely to be representative of the source population with psychosis, given the relatively low proportions of people with these diagnoses who are unknown to secondary care. In line with previous studies from the same region (Fusar-Poli et al., 2020), our cohort contained a relatively high proportion of ethnic minority groups, which is likely driven by the large ethnic minority population in the catchment area. Another possible driving factor is the increased incidence rate of psychosis among ethnic minority groups, which has been reported in a number of studies from the UK (Halvorsrud et al., 2019; Nazroo et al., 2020; Kirkbride et al., 2017).

In line with previous studies based on nationally representative surveys for schizophrenia and schizoaffective disorders in England and Wales (Das-Munshi et al., 2018), our results show that ethnic minority groups with psychosis were, overall, just as likely as their White counterparts to receive antipsychotic medication. However, Black patients were more likely to be prescribed depot antipsychotics, which replicates previous studies (Das-Munshi et al., 2018; Aggarwal et al., 2012). This result is not mediated by level of deprivation, cannabis use, diagnosis or neighbourhood ethnic density. Depot use is generally recommended by clinical guidelines to improve outcomes when patients are noncompliant with medications (Kuipers et al., 2014). This aligns with our results that cannabis use is associated with increased likelihood of recorded depot receipt, given the evidence that cannabis use was associated with noncompliance with antipsychotic medications (Foglia et al., 2017). These results together suggest that, apart from cannabis use, there may be other factors relating to noncompliance with medications and contributing to ethnic inequalities in prescribing depot antipsychotics. Multiple factors can be relevant. First, patients' preferences for treatment, which may be influenced by their beliefs, values and socioeconomic statuses, can affect their compliance with medication (Patel and David, 2005). Second, despite relatively less research from the UK, previous studies from the USA have shown that implicit ethnic bias from providers can lead to problematic perceptions about ethnic minorities, anticipating them being less able to adhere to treatment and more likely to engage in risky health behaviours than their White counterparts, thereby contributing to ethnic disparities in prescribing (Hall et al., 2015). Finally, patient–provider interactions, which may be influenced by implicit ethnic bias from providers and discrepant communication styles between providers and patients from ethnic minority groups, can also have an impact on treatment decisions (Johnson et al., 2004). Thus, further research is needed to clarify the effects of different factors on ethnic inequalities in prescribing antipsychotics.

Consistent with previous studies (Das-Munshi et al., 2018; Copeland et al., 2003; de Freitas et al., 2022), Black patients were less likely to be prescribed a SGA, particularly clozapine. Again, this persisted after adjustment for level of deprivation, cannabis use, diagnosis and neighbourhood ethnic density, suggesting the presence of other contributing factors. A possible explanation is that clinicians may be concerned about physical health complications of clozapine including agranulocytosis, neutropenia and diabetes and increased weight (Farooq et al., 2019), and this may disproportionately affect Black patients, in whom benign ethnic neutropenia (BEN) and diabetes are more common (Copeland et al., 2003). However, a recent literature review has suggested that BEN may be a faulty assumption and stereotype resulting from historically medical racism and scientifically unsound methodologies in early biomedical research (Andreou et al., 2023). The authors found that eight early studies, which established BEN as a legitimate diagnosis, were misleading due to their problematic methodologies such as misuse of ethnicity as a biological or genetic construct without considering its social and political aspects, a lack of clarity around ethnic identification of participants and a lack of considering broader confounding factors (e.g., environmental factors and social determinants of health), thereby creating unjust barriers to accessing clozapine for ethnic minority groups. Thus, justifying under-prescribing of clozapine in Black patients based on BEN concerns without evidence may not only have limited validity, but also can potentially naturalize medical racism and reinforce faulty assumptions in early studies.

Another possible explanation for under-prescribing of clozapine in Black patients is that clozapine requires extensive engagement with regular blood tests and treatment monitoring (Farooq et al., 2019; Gee et al., 2014; Kelly et al., 2018), which can increase the burden for both patients and clinicians, particularly for those in deprived areas with limited resources. This may also disproportionately affect Black patients who were more likely to live in more deprived neighbourhoods. In fact, our results show that living in more deprived neighbourhoods was associated with a lower likelihood in receiving clozapine, which supports the explanation and is also compatible with prior evidence that living in a deprived neighbourhood was associated with clozapine discontinuation (Legge et al., 2016). Nevertheless, as clozapine is generally reserved for patients who have not adequately responded to alternative antipsychotics (Kane et al., 1988), this result may indicate a higher level of under-recognition of treatment resistance and greater numbers of patients who may benefit from clozapine in the Black group.

Our results showed that patients from ethnic minority groups with psychosis were less likely to be prescribed olanzapine. This aligns with the evidence from the USA, where Black patients were found to be less likely to receive olanzapine than White patients (Copeland et al., 2003). However, early studies from the UK did not find an association between ethnicity and prescribing olanzapine (Taylor, 2004). This disparity can be attributed to heterogeneity in populations, definitions of ethnic groups, the time of observation and potentially contributing factors adjusted. In fact, we found that prescribing date was associated with the choice of an antipsychotic, which may reflect the change of clinical guidelines over time. For example, the National Institute for Health and Care Excellence (NICE) recommended SGAs as initial treatments in the early 2000s, while emerging evidence on the risks of SGAs led to a change in the 2009 NICE guidelines with initial choice being driven by side effect profile rather than classification of antipsychotics (Keating et al., 2021). The 2009 update of the Patient Outcome Research Team (PORT) guidelines further specifically excluded olanzapine as an initial treatment option (Buchanan et al., 2010) and other guidelines have also followed (Malhi et al., 2015). Moreover, demographics (e.g., ethnicity distribution) of a local population can change over time, which can be illustrated by the different distributions of different ethnic groups over the date of prescription (Table 1). Thus, time-dependent factors (e.g., date of prescription) are needed to control for potential confounding effects of time on prescribing practices and ethnicity distribution when examining ethnic disparities in antipsychotic prescribing.

Observed disparities in antipsychotic prescribing by ethnicity may not only reflect the variation in biological sensitivities (e.g., different metabolic capacities across racial groups) to antipsychotic medications, but also the role of cultural factors in shaping expectations and response to treatments (Das-Munshi et al., 2018; Taylor, 2004; Bhugra and Bhui, 1999). In addition to demographic and clinical factors that have been widely studied in the literature (Das-Munshi et al., 2018; Connolly et al., 2007; Copeland et al., 2003; Wesley et al., 2021), our models were also adjusted for neighbourhood own-group ethnic density which to some extent reflects individuals' attachment towards to their cultures or ethnic identity (Stronks et al., 2013), as ethnic minorities living in areas with a higher own-group ethnic density may be more oriented towards their cultures of origin (Termorshuizen et al., 2018). Our results suggested that overall ethnic group density might act as an important mediator in linking ethnicity and antipsychotic prescribing. This aligns with recent evidence showing a negative association between own-group ethnic density and dispensing of antipsychotics among the Moroccan- and Turkish-Dutch (Termorshuizen et al., 2018).

Although research on the effect of ethnic density on prescribing is still relatively scant, a possible explanation is that higher-density neighbourhoods may provide more social and family support which can help reduce mental distress (Schofield et al., 2016). Another explanation is that people from collectivistic cultures and with close contact with their families may prefer and benefit from nonpsychopharmacological treatments, such as psychological therapies. For example, prior studies have shown that Asian patients with schizophrenia and schizoaffective disorders were more likely to have been offered family therapy (Das-Munshi et al., 2018). However, own-group ethnic density in the neighbourhood of residence may reflect different factors in different ethnic groups. For example, a higher own-group ethnic density in the Black group might reflect disadvantaged living conditions (Schofield et al., 2016) which may be associated with poorer health outcome and thereby link to increased antipsychotic use. Thus, further research is needed to better understand the role of ethnic density in antipsychotic prescribing.

Apart from these well-known factors, the Covid-19 pandemic appears to contribute to ethnic inequalities in antipsychotic prescribing practices. This may not be surprising as recent studies have shown that the Covid-19 pandemic has deepened existing health inequalities, particularly impacting women, ethnic minorities and those with chronic illnesses (Topriceanu et al., 2021; Feinberg et al., 2022). The pandemic leads to extensive changes in healthcare provision (e.g., prioritising Covid-19 patients and delaying care for non-Covid illnesses) and socioeconomic dynamics (e.g., restrict movement due to lockdown, and disruptions to work and financial statuses) (Topriceanu et al., 2021). Reduced health care access, unstable socioeconomic status, increased psychological distress and decreased community support during lockdown might disproportionately impact ethnic minorities (Topriceanu et al., 2021; Feinberg et al., 2022; Abedi et al., 2021), which can have a negative impact on medication compliance and monitoring routine of adverse effects (Oehl et al., 2000). This then may result in increased prescriptions of depot medications which help to overcome noncompliance and increased prescribing non-clozapine medications that do not need mandatory blood monitoring among ethnic minorities.

### 4.3 Strengths and weaknesses

To the best of our knowledge, this study makes use of the largest sample size to date in a systematic examination of ethnic group differences in use of antipsychotics among patients with psychosis in the UK. The EHR data provided a near-complete representation of patients with psychosis within a large geographical catchment area and represented a broad range of factors which could be controlled for in the analysis, adding to the robustness of associations found. This paper also contributes new approaches for conceptualizing and measuring how ethnicity can influence health inequalities and inform intervention design to eliminate these inequalities.

Limitations of this study include its generalizability, as our cohort was mainly from South London, a region with a high prevalence of psychosis and a high degree of ethnic diversity. Since patient data are protected by restricted access procedures, we cannot test the generalizability of our results in a population from other areas, which requires future investigation. Our data also represent only information from the specialist EHR and we did not attempt to capture prescribing patterns after patients have been discharged to primary care. We did not detect polypharmacy, namely simultaneously prescribing multiple antipsychotics, in our analysis. This however may not significantly alter our results, since polypharmacy is not recommended in UK clinical guidelines (Foster and King, 2020) and SLaM has a considerably lower prevalence (4.8 % reported in (Kadra et al., 2016)) of antipsychotic polypharmacy in comparison to a UK national sample and other US studies (Gallego et al., 2012). As data are taken from the EHR, their integrity is reliant on how information is recorded by clinicians.

In addition, from observed differences, it was not possible to distinguish differences in those offered by clinicians and those chosen by patients.

The quantitative analysis of observational data in this paper did not allow us to 1) clarify the presence of discrimination or racism (a rooted view in explaining health inequalities across ethnic groups (Andreou et al., 2023)), 2) distinguish individual-level discriminatory practices in care provision and system-level discriminatory policies, and 3) measure their impacts on the ethnic disparities in antipsychotic prescribing. These questions need further investigation by incorporating qualitative analysis, such as surveys of both patients and clinicians. We also did not represent use of the Mental Health Act in the data and thus the extent to which prescribing was imposed. Although the use of IMD categories is a widely accepted approach to measure neighbourhood deprivation, it is possible that a postcode area may not be homogeneous and could contain varying levels of deprivation at an individual level. Similar issues also exist when interpreting neighbourhood own-group ethnic density as an indicator of ethnic identity.

### 4.4 Conclusion

Our paper adds to the evidence that there are significant ethnic group inequalities in prescribing of antipsychotics in patients with psychosis. These findings persisted despite controlling for a wide range of potential contributing factors and represent a near-complete picture of the local population with a psychosis diagnosis. We have also demonstrated the utility of using research databases and combining structured data with data gathered using NLP to develop population-level insights. Further research is needed to better understand factors underlying these associations, including patient expectations and perceptions of mental healthcare and clinician factors that may influence their prescribing practices. Also, further development and validation of NLP applications in extracting temporal information of prescriptions from clinical text are needed, so that a full medication history can be retrieved to understand how prescriptions change over time for individual patients and whether patients are managed in a proper pathway in terms of medication use. As the new UK's 2021 Census data have been recently published, another interesting direction is to examine whether using updated Census data, e.g., neighbourhood deprivation and own-group ethnic density, will change our results.

## CRediT authorship contribution statement

Tao Wang: Conceptualization, Methodology, Formal analysis, Visualization, Result interpretation, Writing - original draft, Writing - review & editing.

David Codling: Conceptualization, Methodology, Result Interpretation, Writing - original draft, Writing - review & editing.

Dinesh Bhugra: Conceptualization, Methodology, Writing - review & editing.

Yamiko Msosa: Conceptualization, Writing - review & editing.

Matthew Broadbent: Data extraction, Writing - review & editing.

Rashmi Patel: Writing - review & editing.

Angus Roberts: Writing - review & editing, Supervision.

Philip McGuire: Writing - review & editing, Supervision.

Robert Stewart: Result interpretation, Writing - review & editing, Supervision.

Richard Dobson: Writing - review & editing, Supervision.

Robert Harland: Conceptualization, Result interpretation, Writing - review & editing, Supervision.

## Role of the funding source

The funding bodies had no role in the design of the study, collection and analyses. The views expressed are those of the author(s) and not necessarily those of the NHS, the NIHR or the Department of Health.

## Declaration of competing interest

RS declares research support received in the last 36 months from Janssen, GSK and Takeda, and royalties from Oxford University Press. RP has received grant funding from Janssen and consulting fees from Holmusk, Akrivia Health and Boehringer Ingelheim outside the present study.

## Appendix A Supplementary data

Supplementary material Image 1

## Footnotes

1 https://www.maudsleybrc.nihr.ac.uk/media/463740/applications-library-v21.pdf.

2 https://www.maudsleybrc.nihr.ac.uk/facilities/clinical-record-interactive-search-cris/cris-natural-language-processing/.

3 https://www.ethnicity-facts-figures.service.gov.uk/uk-population-by-ethnicity/national-and-regional-populations/regional-ethnic-diversity/latest.
