# Supplementary material for: Unraveling ethnic disparities in antipsychotic prescribing among patients with psychosis: A retrospective cohort study based on electronic clinical records
Source: Schizophr Res. 2023 Oct;260:168–79. doi: 10.1016/j.schres.2023.08.024 (PMC10881407; doi:10.1016/j.schres.2023.08.024)
Supplement: Supplementary file 1 — Supplementary material [file mmc1.pdf]

Supplemental Information for: Unraveling ethnic disparities in antipsychotic prescribing among patients with psychosis: a retrospective cohort study based on electronic clinical records

Tao Wang, David Codling, Dinesh Bhugra, Yamiko Msosa,
Matthew Broadbent, Rashmi Patel, Angus Roberts,
Philip McGuire, Robert Stewart, Richard Dobson, Robert Harland

Contents

1 Statistics of patient characteristics **2**

2 Sensitivity analyses **2**

List of Figures

S1 Distributions of diagnosis and prescription 2
S2 Mediation analysis of own-group ethnic density in associations between ethnicity and recorded antipsychotic use 5
S3 Mediation analysis of own-group ethnic density in associations between ethnicity and recorded depot prescribing 8
S4 Mediation analysis of own-group ethnic density in associations between ethnicity and recorded SGA prescribing 9
S5 Mediation analysis of own-group ethnic density in associations between ethnicity and recorded olanzapine prescribing 10
S6 Mediation analysis of own-group ethnic density in associations between ethnicity and recorded clozapine prescribing 12

List of Tables

S1 Covariates 3
S2 Prescribing among patients with the latest diagnosis of F20 3
S3 Prescribing among patients with a non-F20 diagnosis 4
S4 Recorded antipsychotic use 4
S5 Effects of own-group ethnic density on antipsychotic use by ethnic groups 6
S6 Prescribing depot antipsychotics 6
S7 Prescribing SGA medications 7
S8 Prescribing olanzapine 7
S9 Prescribing clozapine 11

1 Statistics of patient characteristics

Figure S1 shows the distributions of year of a patient's first F20-F29 diagnosis, illness duration, age at first F20-F29 diagnosis and year of latest antipsychotic prescription recorded. The majority of the first F2* diagnosis information was recorded since 2000 and most patients were diagnosed at age of 20-40 years. Among patients who received an antipsychotic prescription, most of their latest prescriptions were recorded in 2020s.

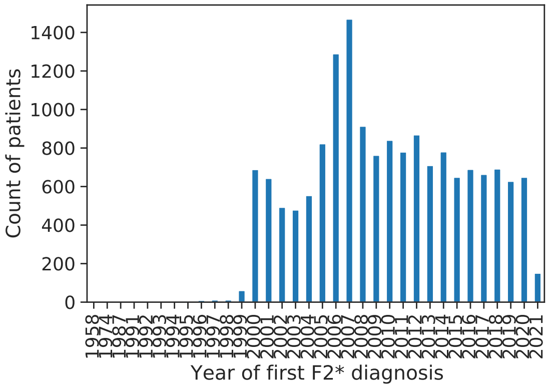

(a)

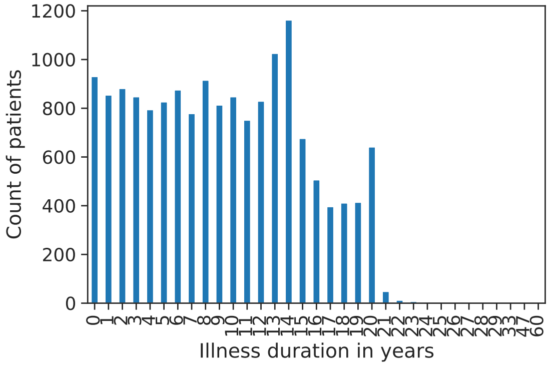

(b)

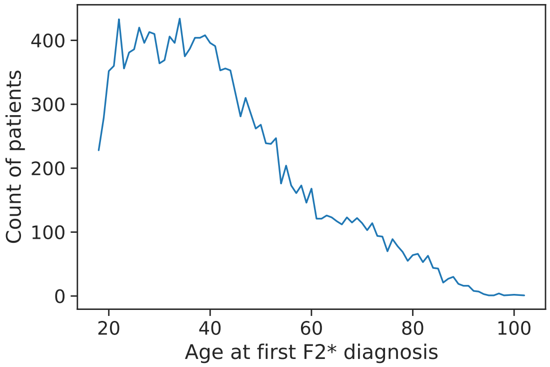

(c)

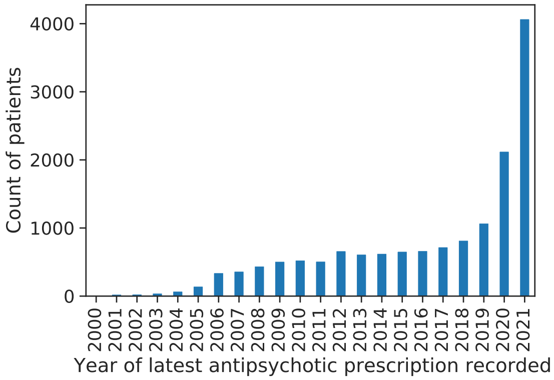

(d)

Figure S1: Distributions of (a) year of a patient's first F2* diagnosis, (b) illness duration from the date of the first F2* diagnoses to 26 March, 2021, i.e., date of data collection, (c) age at first F2* diagnosis and (d) year of latest antipsychotic prescription recorded.

2 Sensitivity analyses

We first conducted sensitivity analyses based on groups with different diagnoses to assess robustness of the associations between explanatory variables and antipsychotic prescribing outcomes. Tables S2 and S3 show the estimated odds ratios in logistic regressions correlating patients characteristics and antipsychotic prescriptions for patients with the latest diagnosis of "F20 – schizophrenia" (the most common diagnosis in our data) and other diagnoses respectively. We find that F20 and non-F20 groups show similar results on the associations between patient ethnicity and prescribing outcomes on receiving antipsychotic prescribing and receiving depot agents. These results are also highly consistent with the main results (Table 3 in the main text). When examining

Table S1: Covariates used in regression analyses.

Variable	Type	Details
Age (in years)	Continuous	Age at the first psychosis diagnosis (identified by ICD-10 codes F2*) calculated based on date of birth and date of the first psychosis diagnosis recorded in patient data
Illness duration (in years)	Continuous	Duration from the date of the first psychosis diagnosis to the date of data collection
Gender	Categorical	From structured field in patient records
Diagnosis	Categorical	ICD-10 codes of latest psychotic disorder diagnosis recorded
EI service	Binary	Latest episodes were managed by any of four early intervention teams in the Trust
Number of inpatient episodes	Continuous	The total inpatient episodes in the Trust
Number of bed days	Continuous	The total bed days in the Trust
Cannabis use	Binary	Evidence of cannabis use extracted from clinical notes using natural language processing
IMD 19 decile	Continuous	Patient postcode of residence is mapped to LSOA (Lower Layer Super Output Area) which is then linked to the 2019 English Index of Multiple Deprivation
Ethnic density	Continuous	Patient postcode of residence is mapped to Geography_code which is then linked to Population by ethnicity and Local Authority from the UK's 2011 Census
Date of prescription	Categorical	Date of latest prescribing recorded, where the date of document recording was used if a prescription was extracted from text document.

Table S2: Odds ratios of logistic regressions correlating patient characteristics and antipsychotic prescriptions among patients with the latest diagnosis of F20.

	Dependent variable:				
	Prescribed an antipsychotic (1)	Prescribed depot (2)	Prescribed SGA (3)	Prescribed olanzapine (4)	Prescribed clozapine (5)
Ethnicity (ref="White")					
Asian	1.02 (0.65, 1.64)	0.89 (0.71, 1.11)	1.21 (0.95, 1.54)	0.81 (0.64, 1.02)	1.15 (0.86, 1.53)
Black	0.89 (0.64, 1.23)	1.24** (1.07, 1.44)	0.98 (0.84, 1.15)	0.88 (0.75, 1.03)	0.65*** (0.53, 0.79)
Mixed	0.62 (0.31, 1.41)	0.80 (0.56, 1.12)	1.24 (0.85, 1.84)	0.63* (0.42, 0.92)	1.42 (0.96, 2.07)
Other	0.57* (0.35, 0.94)	1.04 (0.77, 1.40)	1.16 (0.83, 1.64)	0.95 (0.70, 1.28)	0.92 (0.61, 1.35)
Age	1.02*** (1.01, 1.02)	1.01*** (1.01, 1.02)	0.97*** (0.97, 0.98)	0.99** (0.99, 1.00)	0.96*** (0.96, 0.97)
Illness duration	1.10*** (1.08, 1.12)	1.00 (0.99, 1.01)	0.97*** (0.96, 0.98)	1.01 (1.00, 1.02)	1.02** (1.01, 1.03)
Gender (ref="Male")					
Female	1.14 (0.92, 1.41)	0.91 (0.82, 1.00)	1.07 (0.95, 1.19)	0.87* (0.77, 0.97)	0.95 (0.82, 1.09)
EI service (ref="No")					
Yes	4.08 (1.24, 25.20)	1.02 (0.76, 1.36)	2.16** (1.38, 3.61)	0.82 (0.58, 1.14)	0.20*** (0.10, 0.35)
#Inpatient episodes	8.63*** (5.73, 13.79)	1.14*** (1.11, 1.16)	0.94*** (0.92, 0.96)	0.94*** (0.92, 0.97)	0.93*** (0.91, 0.96)
#Bed days	1.00 (1.00, 1.00)	1.00*** (1.00, 1.00)	1.00 (1.00, 1.00)	1.00 (1.00, 1.00)	1.00*** (1.00, 1.00)
Cannabis use (ref="No")					
Yes	3.35*** (2.59, 4.39)	1.16* (1.04, 1.31)	0.97 (0.85, 1.09)	0.96 (0.84, 1.08)	1.20* (1.03, 1.41)
IMD19 decile	0.96 (0.92, 1.01)	0.97* (0.94, 0.99)	1.01 (0.98, 1.04)	0.99 (0.96, 1.02)	1.07*** (1.04, 1.11)
Ethnic density	0.99*** (0.98, 0.99)	1.00 (1.00, 1.00)	1.00 (1.00, 1.00)	0.99*** (0.99, 1.00)	1.02*** (1.01, 1.02)
Date of prescription (ref="[2000, 2010)")					
[2010, 2020)		0.94 (0.80, 1.09)	1.54*** (1.33, 1.78)	0.96 (0.83, 1.12)	1.18 (0.94, 1.50)
[2020, ∞)		1.69*** (1.44, 1.99)	1.49*** (1.27, 1.76)	0.49*** (0.42, 0.58)	2.84*** (2.26, 3.60)
Constant	2.00* (1.14, 3.51)	0.15*** (0.11, 0.21)	11.52*** (8.19, 16.24)	0.66* (0.47, 0.92)	0.19*** (0.13, 0.30)
Observations	9,416	8,947	8,947	8,947	8,947
Note:					*p<0.05; **p<0.01; ***p<0.001

the results on receiving a specific antipsychotic, such as second-generation antipsychotic (SGA), olanzapine and clozapine, slightly different patterns are observed between F20 and non-F20 groups. Specifically, most ethnic minority groups, except the Mixed and Black groups, are not significantly different from the majority White group in receiving specific antipsychotics among patients diagnosed with F20, while prescribing disparities between ethnic minority groups and the White group are more significant among patients with the non-F20 diagnosis. This result may not be unexpected, as different disorders require different approaches to treatments.

To better understand the relationships between explanatory variables and antipsychotic prescribing outcomes, we then used the one-at-a-time (OAT) approach, i.e., by changing one variable at a time, to examine the effect of an individual variable on the outcomes. Table S4 shows results of regressions correlating ethnicity and recorded antipsychotic use. We found that the associations between ethnicity and antipsychotic use were consistent across Models 1-3, showing that patients from Asian and Black ethnic groups were more likely to have antipsychotic use recorded compared to the White group. However, after controlling for own-group ethnic density in the neighbourhood of residence, patients from most ethnic minority groups, except the Other group, were not significantly different from the White group in receiving an antipsychotic treatment (Model 4), suggesting the presence of own-group ethnic density as a mediator. This was confirmed by a mediation analysis, in which we fitted mediation models [1] by setting own-group ethnic

Table S3: Odds ratios of logistic regressions correlating patient characteristics and antipsychotic prescriptions among patients with a non-F20 latest diagnosis.

	Dependent variable:				
	Prescribed an antipsychotic	Prescribed depot	Prescribed SGA	Prescribed olanzapine	Prescribed clozapine
	(1)	(2)	(3)	(4)	(5)
Ethnicity (ref="White")					
Asian	1.39 (0.95, 2.05)	1.10 (0.77, 1.57)	0.86 (0.59, 1.27)	0.74* (0.58, 0.95)	2.12** (1.21, 3.66)
Black	0.92 (0.72, 1.19)	1.45** (1.15, 1.83)	0.60*** (0.47, 0.77)	0.74*** (0.63, 0.88)	0.99 (0.67, 1.47)
Mixed	1.01 (0.56, 1.97)	1.30 (0.79, 2.08)	0.44** (0.27, 0.74)	0.57** (0.38, 0.83)	1.35 (0.56, 2.95)
Other	0.71 (0.49, 1.01)	1.05 (0.70, 1.55)	0.82 (0.54, 1.28)	0.73* (0.56, 0.95)	1.97* (1.02, 3.68)
Age	1.01*** (1.01, 1.02)	1.00 (1.00, 1.01)	0.99*** (0.98, 0.99)	0.99* (0.99, 1.00)	0.97*** (0.96, 0.98)
Illness duration	1.05*** (1.03, 1.07)	1.00 (0.98, 1.01)	1.00 (0.98, 1.02)	0.99 (0.98, 1.00)	1.01 (0.99, 1.04)
Gender (ref="Male")					
Female	1.17 (0.98, 1.39)	0.80** (0.68, 0.94)	1.06 (0.90, 1.25)	0.82*** (0.72, 0.92)	1.10 (0.86, 1.41)
Diagnosis (ref="F29")					
F22	0.44*** (0.34, 0.58)	0.89 (0.64, 1.22)	1.14 (0.81, 1.61)	0.63*** (0.50, 0.78)	1.14 (0.52, 2.35)
F23	0.57*** (0.45, 0.72)	0.43*** (0.30, 0.60)	1.22 (0.89, 1.69)	1.40*** (1.18, 1.65)	0.32* (0.11, 0.77)
F25	2.66*** (1.94, 3.67)	2.34*** (1.88, 2.91)	0.41*** (0.32, 0.53)	0.72*** (0.61, 0.86)	5.56*** (3.65, 8.73)
Other	0.75 (0.54, 1.06)	0.69 (0.42, 1.08)	1.05 (0.68, 1.70)	1.01 (0.77, 1.31)	0.91 (0.31, 2.22)
EI service (ref="No")					
Yes	2.41*** (1.83, 3.20)	0.53*** (0.40, 0.70)	2.53*** (1.80, 3.64)	0.93 (0.79, 1.09)	0.26*** (0.12, 0.51)
#Inpatient episodes	5.26*** (4.16, 6.70)	1.14*** (1.11, 1.18)	0.93*** (0.90, 0.96)	0.95** (0.92, 0.99)	0.92*** (0.88, 0.96)
#Bed days	1.00 (1.00, 1.00)	1.00 (1.00, 1.00)	1.00 (1.00, 1.00)	1.00 (1.00, 1.00)	1.00*** (1.00, 1.00)
Cannabis use (ref="No")					
Yes	1.66*** (1.36, 2.03)	1.27* (1.05, 1.52)	1.11 (0.91, 1.34)	0.93 (0.82, 1.06)	1.22 (0.90, 1.66)
IMD19 decile	1.03 (0.99, 1.08)	1.00 (0.96, 1.05)	0.99 (0.95, 1.03)	1.01 (0.98, 1.04)	1.04 (0.98, 1.11)
Ethnic density	0.99* (0.99, 1.00)	1.00 (1.00, 1.01)	0.99* (0.99, 1.00)	0.99** (0.99, 1.00)	1.02*** (1.02, 1.03)
Date of prescription (ref="[2000, 2010)")					
[2010, 2020)		1.55** (1.13, 2.18)	1.31* (1.01, 1.70)	0.80* (0.66, 0.96)	1.29 (0.76, 2.32)
[2020, ∞)		2.22*** (1.59, 3.13)	1.11 (0.84, 1.48)	0.63*** (0.51, 0.78)	3.31*** (1.94, 5.96)
Constant	1.33 (0.83, 2.13)	0.05*** (0.03, 0.08)	20.35*** (11.81, 35.34)	1.31 (0.90, 1.92)	0.01*** (0.004, 0.03)
Observations	6,892	6,158	6,158	6,158	6,158
Note:					*p<0.05; **p<0.01; ***p<0.001

density as a mediator, ethnicity as a treatment status, and receipt of any antipsychotic medication as the outcome variable, conditional on other control variables. We found that, in most cases, the estimated average causal mediation effects (ACMEs) were statistically significantly different from zero but the estimated average direct effects (ADEs) were not (Figure S2). These results indicate that ethnic minority groups may have lower own-group ethnic density in neighbourhood, which in turn leads to these groups being more likely to receive antipsychotic prescribing. This finding aligns with previous studies, in which neighbourhood ethnic density was found to be associated with lower dispensing of antipsychotics among the Moroccan-Dutch [2].

Table S4: Odds ratios of logistic regressions correlating patient characteristics and prescribing any antipsychotic.

	Dependent variable:			
	Prescribed an antipsychotic			
	(1)	(2)	(3)	(4)
Ethnicity (ref="White")				
Asian	1.69*** (1.32, 2.19)	1.83*** (1.43, 2.39)	1.83*** (1.42, 2.38)	1.23 (0.92, 1.66)
Black	1.31*** (1.13, 1.51)	1.29*** (1.11, 1.50)	1.27** (1.10, 1.48)	0.90 (0.74, 1.10)
Mixed	1.35 (0.87, 2.17)	1.30 (0.84, 2.10)	1.29 (0.84, 2.10)	0.85 (0.53, 1.40)
Other	0.92 (0.72, 1.17)	0.97 (0.76, 1.24)	0.96 (0.75, 1.23)	0.63** (0.47, 0.84)
Age	1.01*** (1.01, 1.01)	1.02*** (1.01, 1.02)	1.02*** (1.01, 1.02)	1.02*** (1.01, 1.02)
Illness duration	1.07*** (1.06, 1.09)	1.08*** (1.06, 1.09)	1.08*** (1.06, 1.09)	1.07*** (1.06, 1.09)
Gender (ref="Male")				
Female	1.02 (0.90, 1.17)	1.18* (1.03, 1.35)	1.18* (1.03, 1.35)	1.16* (1.02, 1.33)
Diagnosis (ref="F29")				
F20	1.77*** (1.44, 2.18)	1.80*** (1.45, 2.21)	1.80*** (1.46, 2.21)	1.86*** (1.51, 2.29)
F22	0.45*** (0.35, 0.58)	0.44*** (0.34, 0.57)	0.44*** (0.35, 0.57)	0.45*** (0.35, 0.58)
F23	0.52*** (0.41, 0.66)	0.54*** (0.42, 0.68)	0.54*** (0.43, 0.69)	0.54*** (0.43, 0.69)
25	2.54*** (1.86, 3.49)	2.54*** (1.87, 3.50)	2.55*** (1.87, 3.52)	2.69*** (1.97, 3.71)
Other	0.73 (0.53, 1.03)	0.73 (0.52, 1.02)	0.73 (0.52, 1.02)	0.72 (0.52, 1.02)
EI service (ref="No")				
Yes	2.74*** (2.11, 3.60)	2.52*** (1.94, 3.32)	2.52*** (1.93, 3.32)	2.45*** (1.88, 3.22)
#Inpatient episodes	6.85*** (5.63, 8.41)	6.16*** (5.07, 7.57)	6.15*** (5.06, 7.55)	6.02*** (4.95, 7.41)
#Bed days	1.00 (1.00, 1.00)	1.00 (1.00, 1.00)	1.00 (1.00, 1.00)	1.00 (1.00, 1.00)
Cannabis use (ref="No")				
Yes		2.22*** (1.91, 2.60)	2.22*** (1.90, 2.59)	2.20*** (1.89, 2.57)
IMD19 decile			0.99 (0.96, 1.02)	1.00 (0.97, 1.03)
Ethnic density				0.99*** (0.99, 0.99)
Constant	1.41* (1.08, 1.86)	0.74* (0.54, 1.00)	0.79 (0.56, 1.10)	1.20 (0.83, 1.74)
Observations	16,308	16,308	16,308	16,308
Note:				*p<0.05; **p<0.01; ***p<0.001

To examine whether the associations between own-group ethnic density and antipsychotic use

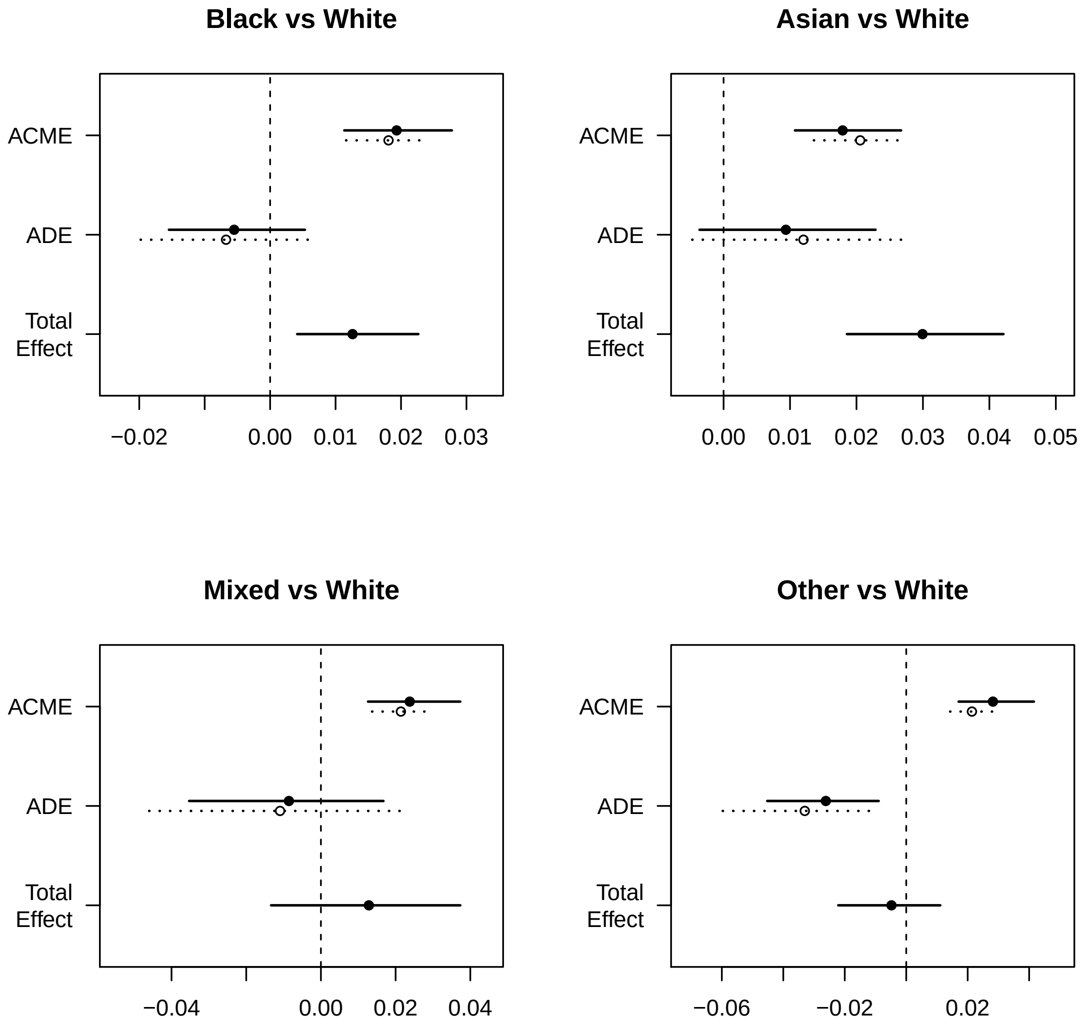

Figure S2: Visualization of mediation analysis results (average causal mediation effects (ACMEs) and average direct effects (ADEs)) for own-group ethnic density as a mediator in examining associations between ethnicity and recorded antipsychotic use, controlling for all covariates in Model 5 of Table S4. The dashed line represents the control group, namely the White group, and solid line represents a treated group, namely an ethnic minority group as stated in the title of each plot.

have similar patterns across ethnic groups, a logistic regression model was used for each ethnic group to examine the association between own-group ethnic density and recorded antipsychotic use, adjusted on other control variables (Table S5). Mixed results were observed, where a higher own-group ethnic density in neighborhood was associated with decreased antipsychotic use in the White and Other groups (Models 1 and 5), while a positive association between ethnic density and antipsychotic use was found in the Black group (Model 2). This suggests that neighbourhood ethnic density might not only reflect increased social support which is often considered to be associated with better mental health [2], but also other factors that may be associated with poorer

health outcome and thereby link to increased antipsychotic use, such as disadvantaged living conditions in some ethnic groups [3].

Table S5: Odds ratios of logistic regressions correlating patient characteristics and prescribing any antipsychotic by ethnic groups.

	Dependent variable:				
	Prescribed an antipsychotic				
	(1)	(2)	(3)	(4)	(5)
	White	Black	Asian	Mixed	Other
Age	1.02*** (1.01, 1.03)	1.00 (1.00, 1.01)	1.02* (1.00, 1.03)	1.02 (0.98, 1.06)	1.00 (0.98, 1.02)
Illness duration	1.07*** (1.05, 1.09)	1.09*** (1.06, 1.11)	1.06* (1.01, 1.12)	1.18** (1.05, 1.34)	0.99 (0.94, 1.04)
Gender (ref="Male")					
Female	1.10 (0.91, 1.33)	1.36* (1.04, 1.77)	0.84 (0.51, 1.40)	0.80 (0.25, 2.58)	1.30 (0.80, 2.14)
Diagnosis (ref="F29")					
F20	1.79*** (1.32, 2.41)	2.16*** (1.45, 3.17)	2.37* (1.11, 4.94)	1.33 (0.33, 5.10)	2.15* (1.02, 4.44)
F22	0.49*** (0.34, 0.71)	0.30*** (0.19, 0.47)	0.88 (0.35, 2.21)	0.40 (0.06, 2.66)	0.65 (0.28, 1.51)
F23	0.57** (0.40, 0.81)	0.43*** (0.29, 0.66)	0.84 (0.37, 1.96)	0.42 (0.06, 3.73)	0.60 (0.28, 1.29)
F25	3.03*** (1.99, 4.68)	2.10* (1.16, 3.98)	4.21* (1.27, 19.19)	15,343,481.00 (0.00, 32,332,207,820.00)	2.15 (0.76, 6.75)
Other	0.78 (0.49, 1.25)	0.74 (0.40, 1.44)	0.78 (0.23, 3.12)	0.48 (0.04, 11.82)	0.63 (0.21, 1.95)
EI service (ref="No")					
Yes	2.78*** (1.83, 4.37)	2.06** (1.35, 3.21)	3.42* (1.32, 10.71)	3.82 (0.89, 20.99)	1.64 (0.71, 4.15)
#Inpatient episodes	5.70*** (4.12, 8.06)	5.57*** (4.14, 7.69)	3.81** (1.56, 9.62)	5.40 (0.64, 54.79)	7.05*** (3.25, 17.23)
#Bed days	1.00 (1.00, 1.00)	1.00 (1.00, 1.00)	1.01 (1.00, 1.03)	1.01 (1.00, 1.10)	1.00 (1.00, 1.01)
Cannabis use (ref="No")					
Yes	2.46*** (1.98, 3.06)	2.04*** (1.53, 2.72)	1.74 (0.96, 3.28)	2.78 (0.87, 9.42)	2.57*** (1.51, 4.52)
IMD19 decile	1.00 (0.96, 1.04)	1.04 (0.97, 1.12)	0.96 (0.85, 1.09)	1.20 (0.93, 1.59)	1.03 (0.91, 1.17)
Ethnic density	0.99*** (0.99, 0.99)	1.12*** (1.08, 1.16)	1.05 (0.97, 1.16)	0.95 (0.46, 1.94)	0.72* (0.55, 0.95)
Constant	1.04 (0.65, 1.67)	0.58 (0.31, 1.10)	1.30 (0.39, 4.39)	0.30 (0.02, 3.76)	4.33* (1.15, 16.92)
Observations	7,029	6,654	1,356	431	838
Note:					*p<0.05; **p<0.01; ***p<0.001

Similar mediation effects of own-group ethnic density were found in the analyses for other prescribing outcomes, including receipt of olanzapine or clozapine (Tables S8-S9 and Figures S5-S6). However, ethnic density was not significantly associated with receipt of a depot agent or SGA medications and the associations between ethnicity and these prescribing outcomes were much consistent across different model specifications (Tables S6-S7 and Figures S3-S4).

Table S6: Odds ratios of logistic regressions correlating patient characteristics and prescribing depot antipsychotics.

	Dependent variable:				
	Prescribed depot				
	(1)	(2)	(3)	(4)	(5)
Ethnicity (ref="White")					
Asian	0.91 (0.77, 1.06)	0.94 (0.80, 1.09)	0.93 (0.79, 1.09)	0.93 (0.77, 1.12)	0.94 (0.78, 1.14)
Black	1.32*** (1.21, 1.44)	1.31*** (1.20, 1.43)	1.29*** (1.18, 1.41)	1.29*** (1.14, 1.46)	1.29*** (1.14, 1.47)
Mixed	0.94 (0.72, 1.20)	0.92 (0.71, 1.19)	0.92 (0.71, 1.18)	0.92 (0.69, 1.21)	0.93 (0.70, 1.22)
Other	1.01 (0.82, 1.24)	1.01 (0.82, 1.24)	1.01 (0.81, 1.24)	1.01 (0.80, 1.27)	1.02 (0.81, 1.29)
Age	1.01*** (1.00, 1.01)	1.01*** (1.01, 1.01)	1.01*** (1.01, 1.01)	1.01*** (1.01, 1.01)	1.01*** (1.01, 1.01)
Illness duration	1.00 (0.99, 1.01)	1.00 (0.99, 1.01)	1.00 (0.99, 1.01)	1.00 (0.99, 1.01)	1.00 (0.99, 1.01)
Gender (ref="Male")					
Female	0.84*** (0.78, 0.92)	0.88** (0.81, 0.96)	0.89** (0.81, 0.97)	0.89** (0.81, 0.97)	0.87** (0.80, 0.95)
Diagnosis (ref="F29")					
F20	2.79*** (2.35, 3.33)	2.77*** (2.33, 3.31)	2.78*** (2.34, 3.31)	2.78*** (2.34, 3.31)	2.88*** (2.42, 3.44)
F22	0.74* (0.55, 0.99)	0.73* (0.54, 0.98)	0.73* (0.54, 0.98)	0.73* (0.54, 0.98)	0.77 (0.57, 1.03)
F23	0.36*** (0.26, 0.51)	0.37*** (0.26, 0.51)	0.37*** (0.26, 0.51)	0.37*** (0.26, 0.51)	0.43*** (0.31, 0.60)
F25	2.36*** (1.94, 2.87)	2.33*** (1.92, 2.83)	2.33*** (1.92, 2.84)	2.33*** (1.92, 2.84)	2.41*** (1.98, 2.94)
Other	0.61* (0.38, 0.95)	0.61* (0.38, 0.96)	0.61* (0.38, 0.96)	0.61* (0.38, 0.96)	0.67 (0.41, 1.04)
EI service (ref="No")					
Yes	0.76** (0.63, 0.93)	0.75** (0.61, 0.91)	0.75** (0.61, 0.91)	0.75** (0.61, 0.91)	0.73** (0.60, 0.89)
#Inpatient episodes	1.17*** (1.16, 1.20)	1.16*** (1.14, 1.18)	1.16*** (1.14, 1.18)	1.16*** (1.14, 1.18)	1.14*** (1.12, 1.16)
#Bed days	1.00*** (1.00, 1.00)	1.00*** (1.00, 1.00)	1.00*** (1.00, 1.00)	1.00*** (1.00, 1.00)	1.00*** (1.00, 1.00)
Cannabis use (ref="No")					
Yes		1.30*** (1.18, 1.43)	1.30*** (1.18, 1.43)	1.30*** (1.18, 1.43)	1.19*** (1.08, 1.31)
IMD19 decile			0.98* (0.95, 1.00)	0.98* (0.95, 1.00)	0.98 (0.96, 1.00)
Ethnic density				1.00 (1.00, 1.00)	1.00 (1.00, 1.00)
Date of prescription (ref="[2000, 2010)")					
[2010, 2020)					1.04 (0.91, 1.19)
[2020, ∞)					1.74*** (1.51, 2.01)
Constant	0.09*** (0.07, 0.11)	0.07*** (0.05, 0.09)	0.07*** (0.06, 0.10)	0.07*** (0.06, 0.10)	0.05*** (0.04, 0.07)
Observations	15,105	15,105	15,105	15,105	15,105
Note:					*p<0.05; **p<0.01; ***p<0.001

Table S7: Odds ratios of logistic regressions correlating patient characteristics and prescribing SGA medications.

	Dependent variable:				
	Prescribed SGA				
	(1)	(2)	(3)	(4)	(5)
Ethnicity (ref="White")					
Asian	1.20* (1.01, 1.43)	1.21* (1.02, 1.44)	1.21* (1.02, 1.44)	1.12 (0.91, 1.37)	1.09 (0.89, 1.34)
Black	0.92 (0.84, 1.02)	0.92 (0.84, 1.01)	0.92 (0.84, 1.01)	0.86* (0.75, 0.98)	0.85* (0.74, 0.97)
Mixed	0.97 (0.74, 1.29)	0.97 (0.74, 1.29)	0.97 (0.74, 1.29)	0.89 (0.66, 1.21)	0.87 (0.65, 1.18)
Other	1.18 (0.94, 1.51)	1.19 (0.94, 1.51)	1.19 (0.94, 1.51)	1.09 (0.84, 1.42)	1.05 (0.81, 1.37)
Age	0.98*** (0.97, 0.98)	0.98*** (0.97, 0.98)	0.98*** (0.97, 0.98)	0.98*** (0.97, 0.98)	0.98*** (0.97, 0.98)
Illness duration	0.98*** (0.97, 0.99)	0.98*** (0.97, 0.99)	0.98*** (0.97, 0.99)	0.98*** (0.97, 0.99)	0.98*** (0.97, 0.99)
Gender (ref="Male")					
Female	1.06 (0.97, 1.16)	1.08 (0.98, 1.18)	1.08 (0.98, 1.18)	1.07 (0.98, 1.18)	1.06 (0.97, 1.16)
Diagnosis (ref="F29")					
F20	0.45*** (0.37, 0.56)	0.45*** (0.37, 0.55)	0.45*** (0.37, 0.55)	0.45*** (0.37, 0.56)	0.46*** (0.38, 0.57)
F22	1.58** (1.15, 2.17)	1.57** (1.15, 2.16)	1.57** (1.15, 2.16)	1.58** (1.15, 2.16)	1.62** (1.19, 2.23)
F23	1.31 (0.96, 1.80)	1.31 (0.96, 1.80)	1.31 (0.96, 1.80)	1.32 (0.97, 1.81)	1.38* (1.01, 1.89)
F25	0.44*** (0.35, 0.55)	0.44*** (0.35, 0.55)	0.44*** (0.35, 0.55)	0.44*** (0.35, 0.55)	0.45*** (0.36, 0.56)
Other	1.21 (0.78, 1.95)	1.21 (0.78, 1.95)	1.21 (0.78, 1.95)	1.21 (0.78, 1.95)	1.27 (0.82, 2.05)
EI service (ref="No")					
Yes	2.22*** (1.69, 2.96)	2.21*** (1.68, 2.95)	2.21*** (1.68, 2.95)	2.20*** (1.67, 2.94)	2.20*** (1.67, 2.94)
#Inpatient episodes	0.94*** (0.93, 0.96)	0.94*** (0.92, 0.96)	0.94*** (0.92, 0.96)	0.94*** (0.92, 0.96)	0.94*** (0.92, 0.95)
#Bed days	1.00 (1.00, 1.00)	1.00 (1.00, 1.00)	1.00 (1.00, 1.00)	1.00 (1.00, 1.00)	1.00 (1.00, 1.00)
Cannabis use (ref="No")					
Yes		1.06 (0.96, 1.17)	1.06 (0.96, 1.17)	1.06 (0.96, 1.17)	1.02 (0.92, 1.13)
IMD19 decile			1.00 (0.98, 1.02)	1.00 (0.98, 1.03)	1.00 (0.98, 1.03)
Ethnic density				1.00 (0.99, 1.00)	1.00 (0.99, 1.00)
Date of prescription (ref="[2000, 2010)")					
[2010, 2020)					1.47*** (1.30, 1.67)
[2020, ∞)					1.37*** (1.19, 1.57)
Constant	29.14*** (22.65, 37.75)	27.58*** (21.06, 36.35)	27.46*** (20.62, 36.78)	29.87*** (21.97, 40.84)	22.17*** (16.02, 30.84)
Observations	15,105	15,105	15,105	15,105	15,103
Note:					*p<0.05; **p<0.01; ***p<0.001

Table S8: Odds ratios of logistic regressions correlating patient characteristics and prescribing olanzapine.

	Dependent variable:				
	Prescribed olanzapine				
	(1)	(2)	(3)	(4)	(5)
Ethnicity (ref="White")					
Asian	0.96 (0.84, 1.11)	0.95 (0.82, 1.09)	0.95 (0.82, 1.09)	0.78** (0.66, 0.92)	0.78** (0.66, 0.92)
Black	0.95 (0.88, 1.04)	0.96 (0.88, 1.04)	0.95 (0.88, 1.04)	0.81*** (0.72, 0.91)	0.82*** (0.73, 0.92)
Mixed	0.73* (0.56, 0.94)	0.74* (0.57, 0.95)	0.74* (0.57, 0.95)	0.60*** (0.46, 0.79)	0.60*** (0.46, 0.79)
Other	1.01 (0.85, 1.20)	1.01 (0.84, 1.20)	1.01 (0.84, 1.20)	0.82 (0.68, 1.00)	0.82* (0.67, 0.99)
Age	1.00 (0.99, 1.00)	1.00** (0.99, 1.00)	1.00** (0.99, 1.00)	1.00** (0.99, 1.00)	0.99*** (0.99, 1.00)
Illness duration	1.00 (0.99, 1.01)	1.00 (0.99, 1.01)	1.00 (0.99, 1.01)	1.00 (0.99, 1.01)	1.00 (0.99, 1.01)
Gender (ref="Male")					
Female	0.86*** (0.79, 0.93)	0.84*** (0.77, 0.91)	0.84*** (0.77, 0.91)	0.83*** (0.77, 0.91)	0.84*** (0.78, 0.92)
Diagnosis (ref="F29")					
F20	0.59*** (0.51, 0.67)	0.59*** (0.52, 0.67)	0.59*** (0.52, 0.67)	0.60*** (0.52, 0.68)	0.58*** (0.51, 0.67)
F22	0.62*** (0.50, 0.76)	0.63*** (0.51, 0.77)	0.63*** (0.51, 0.77)	0.63*** (0.51, 0.77)	0.59*** (0.48, 0.73)
F23	1.48*** (1.26, 1.73)	1.47*** (1.25, 1.72)	1.47*** (1.25, 1.73)	1.47*** (1.26, 1.73)	1.29** (1.10, 1.52)
F25	0.70*** (0.59, 0.82)	0.70*** (0.60, 0.82)	0.70*** (0.60, 0.82)	0.71*** (0.61, 0.84)	0.70*** (0.60, 0.82)
Other	1.03 (0.80, 1.33)	1.03 (0.79, 1.33)	1.03 (0.79, 1.33)	1.04 (0.80, 1.34)	0.96 (0.74, 1.24)
EI service (ref="No")					
Yes	0.91 (0.79, 1.05)	0.92 (0.80, 1.06)	0.92 (0.80, 1.06)	0.91 (0.79, 1.05)	0.93 (0.81, 1.07)
#Inpatient episodes	0.92*** (0.90, 0.94)	0.93*** (0.91, 0.95)	0.93*** (0.91, 0.95)	0.92*** (0.90, 0.94)	0.95*** (0.93, 0.97)
#Bed days	1.00 (1.00, 1.00)	1.00 (1.00, 1.00)	1.00 (1.00, 1.00)	1.00 (1.00, 1.00)	1.00 (1.00, 1.00)
Cannabis use (ref="No")					
Yes		0.88** (0.81, 0.96)	0.88** (0.81, 0.96)	0.88** (0.81, 0.96)	0.95 (0.86, 1.04)
IMD19 decile			1.00 (0.98, 1.02)	1.00 (0.98, 1.03)	1.00 (0.98, 1.02)
Ethnic density				0.99*** (0.99, 1.00)	0.99*** (0.99, 1.00)
Date of prescription (ref="[2000, 2010)")					
[2010, 2020)					0.90 (0.80, 1.00)
[2020, ∞)					0.57*** (0.50, 0.65)
Constant	0.63*** (0.53, 0.76)	0.72*** (0.59, 0.87)	0.72** (0.58, 0.89)	0.87 (0.69, 1.10)	1.20 (0.93, 1.55)
Observations	15,105	15,105	15,105	15,105	15,105
Note:					*p<0.05; **p<0.01; ***p<0.001

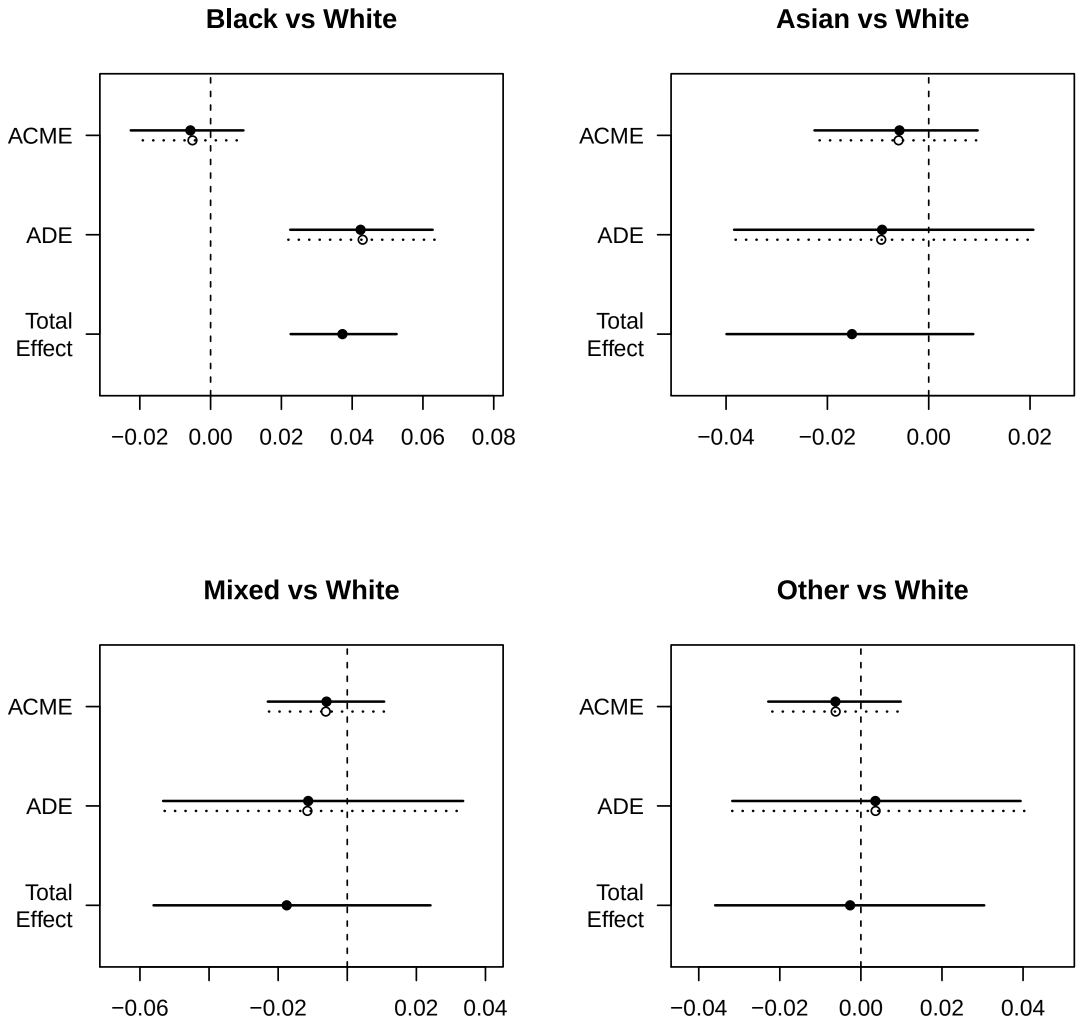

Figure S3: Visualization of mediation analysis results (average causal mediation effects (ACMEs) and average direct effects (ADEs)) for own-group ethnic density as a mediator in examining associations between ethnicity and recorded depot prescribing, controlling for all covariates in Model 4 of Table S6. The dashed line represents the control group, namely the White group, and solid line represents a treated group, namely an ethnic minority group as stated in the title of each plot.

References

[1] D. Tingley, T. Yamamoto, K. Hirose, L. Keele, K. Imai, Mediation: R package for causal mediation analysis (2014).

[2] F. Termorshuizen, E. R. Heerdink, J.-P. Selten, The impact of ethnic density on dispensing of antipsychotic and antidepressant medication among immigrants in the netherlands, Social Science & Medicine 211 (2018) 87–94.

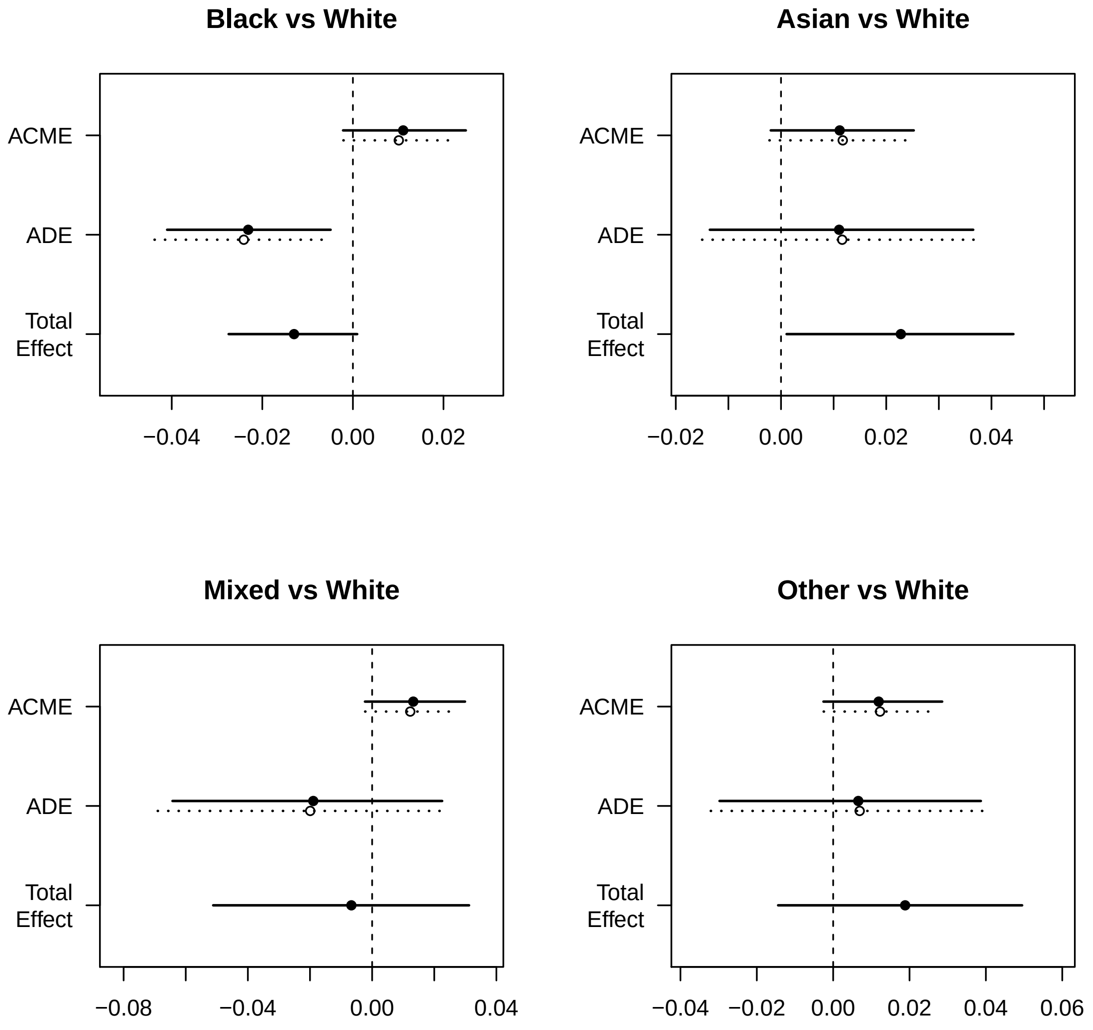

Figure S4: Visualization of mediation analysis results (average causal mediation effects (ACMEs) and average direct effects (ADEs)) for own-group ethnic density as a mediator in examining associations between ethnicity and recorded SGA prescribing, controlling for all covariates in Model 5 of Table S7. The dashed line represents the control group, namely the White group, and solid line represents a treated group, namely an ethnic minority group as stated in the title of each plot.

[3] P. Schofield, J. Das-Munshi, L. Bécares, C. Morgan, V. Bhavsar, M. Hotopf, S. L. Hatch, Minority status and mental distress: a comparison of group density effects, Psychological medicine 46 (14) (2016) 3051–3059.

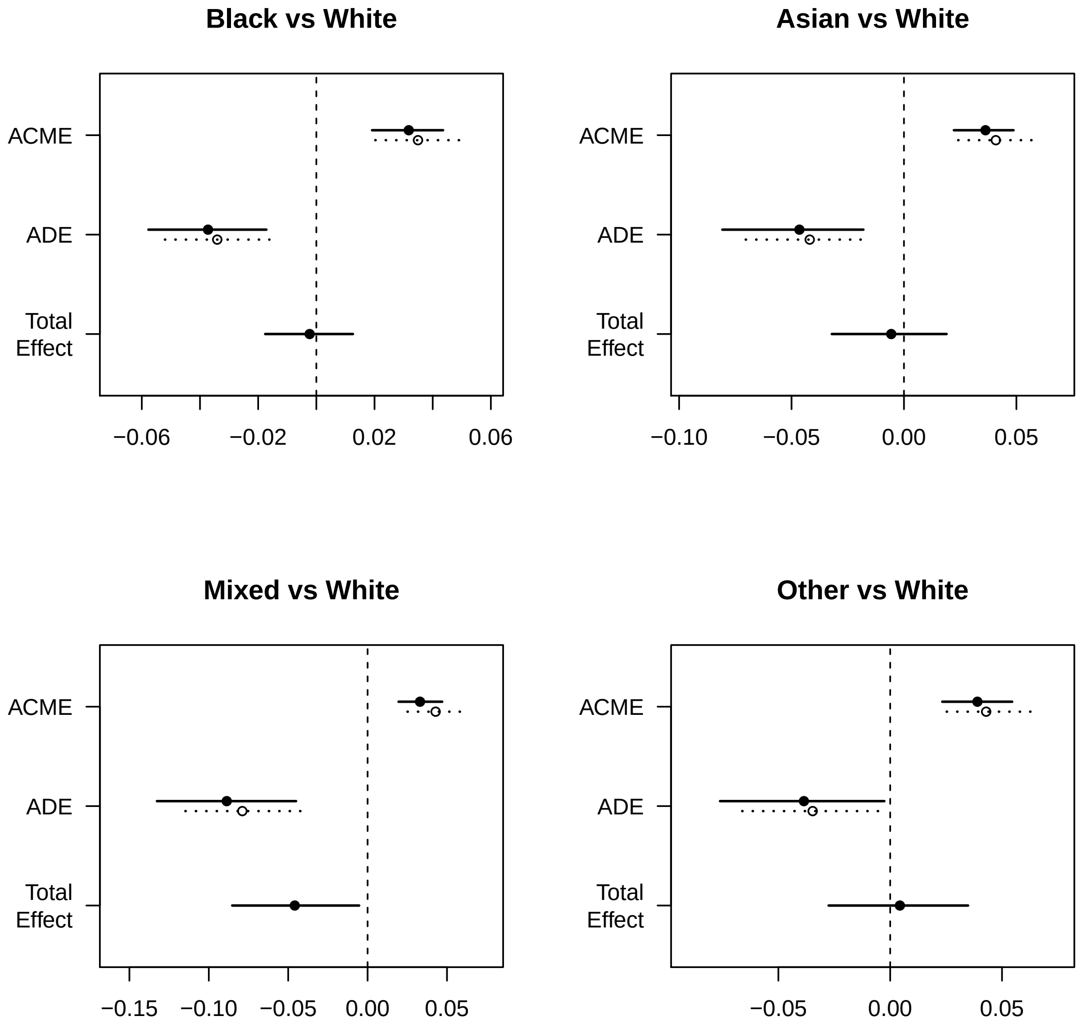

Figure S5: Visualization of mediation analysis results (average causal mediation effects (ACMEs) and average direct effects (ADEs)) for own-group ethnic density as a mediator in examining associations between ethnicity and recorded olanzapine prescribing, controlling for all covariates in Model 5 of Table S8. The dashed line represents the control group, namely the White group, and solid line represents a treated group, namely an ethnic minority group as stated in the title of each plot.

Table S9: Odds ratios of logistic regressions correlating patient characteristics and prescribing clozapine.

	Dependent variable:				
	Prescribed clozapine				
	(1)	(2)	(3)	(4)	(5)
Ethnicity (ref="White")					
Asian	0.63^{***} (0.51, 0.78)	0.67^{***} (0.54, 0.82)	0.68^{***} (0.55, 0.84)	1.20 (0.94, 1.54)	1.31^{*} (1.02, 1.69)
Black	0.41^{***} (0.36, 0.46)	0.40^{***} (0.36, 0.46)	0.42^{***} (0.37, 0.48)	0.69^{***} (0.58, 0.82)	0.71^{***} (0.60, 0.85)
Mixed	0.72^{*} (0.53, 0.98)	0.71^{*} (0.52, 0.96)	0.72^{*} (0.52, 0.97)	1.30 (0.92, 1.81)	1.42^{*} (1.00, 1.99)
Other	0.54^{***} (0.40, 0.72)	0.55^{***} (0.40, 0.73)	0.56^{***} (0.41, 0.75)	1.03 (0.73, 1.42)	1.12 (0.79, 1.55)
Age	0.96^{***} (0.95, 0.96)	0.96^{***} (0.96, 0.97)	0.96^{***} (0.96, 0.97)	0.96^{***} (0.96, 0.97)	0.96^{***} (0.96, 0.97)
Illness duration	1.02^{**} (1.01, 1.03)	1.02^{**} (1.01, 1.03)	1.02^{***} (1.01, 1.03)	1.02^{***} (1.01, 1.03)	1.02^{***} (1.01, 1.03)
Gender (ref="Male")					
Female	0.94 (0.84, 1.06)	1.00 (0.89, 1.13)	1.00 (0.88, 1.12)	1.01 (0.90, 1.14)	0.98 (0.87, 1.11)
Diagnosis (ref="F29")					
F20	7.74^{***} (5.41, 11.49)	7.68^{***} (5.36, 11.40)	7.62^{***} (5.32, 11.32)	7.25^{***} (5.06, 10.77)	7.98^{***} (5.55, 11.89)
F22	1.15 (0.54, 2.27)	1.14 (0.54, 2.25)	1.13 (0.53, 2.23)	1.08 (0.51, 2.14)	1.28 (0.60, 2.54)
F23	0.23^{**} (0.08, 0.54)	0.23^{**} (0.08, 0.55)	0.22^{**} (0.08, 0.53)	0.22^{**} (0.07, 0.52)	0.31^{*} (0.11, 0.74)
F25	6.04^{***} (4.15, 9.11)	5.94^{***} (4.07, 8.95)	5.85^{***} (4.01, 8.82)	5.45^{***} (3.73, 8.23)	5.96^{***} (4.07, 9.01)
Other	0.75 (0.25, 1.80)	0.76 (0.26, 1.83)	0.76 (0.26, 1.82)	0.71 (0.24, 1.71)	0.89 (0.30, 2.15)
EI service (ref="No")					
Yes	0.22^{***} (0.13, 0.33)	0.21^{***} (0.13, 0.33)	0.21^{***} (0.13, 0.33)	0.22^{***} (0.14, 0.35)	0.22^{***} (0.13, 0.35)
#Inpatient episodes	0.97^{***} (0.95, 0.98)	0.96^{***} (0.94, 0.97)	0.96^{***} (0.94, 0.98)	0.96^{***} (0.94, 0.98)	0.93^{***} (0.91, 0.95)
#Bed days	1.00^{***} (1.00, 1.00)	1.00^{***} (1.00, 1.00)	1.00^{***} (1.00, 1.00)	1.00^{***} (1.00, 1.00)	1.00^{***} (1.00, 1.00)
Cannabis use (ref="No")					
Yes		1.43^{***} (1.25, 1.63)	1.44^{***} (1.26, 1.65)	1.44^{***} (1.26, 1.65)	1.21^{**} (1.05, 1.39)
IMD19 decile			1.08^{***} (1.05, 1.11)	1.06^{***} (1.03, 1.09)	1.07^{***} (1.04, 1.10)
Ethnic density				1.01^{***} (1.01, 1.02)	1.02^{***} (1.01, 1.02)
Date of prescription (ref="[2000, 2010)")					
[2010, 2020)					1.19 (0.97, 1.48)
[2020, ∞)					2.90^{***} (2.35, 3.61)
Constant	0.14^{***} (0.09, 0.21)	0.10^{***} (0.06, 0.15)	0.07^{***} (0.05, 0.11)	0.04^{***} (0.03, 0.07)	0.02^{***} (0.01, 0.03)
Observations	15,105	15,105	15,105	15,105	15,105

Note: $^{*}p<0.05$; $^{**}p<0.01$; $^{***}p<0.001$

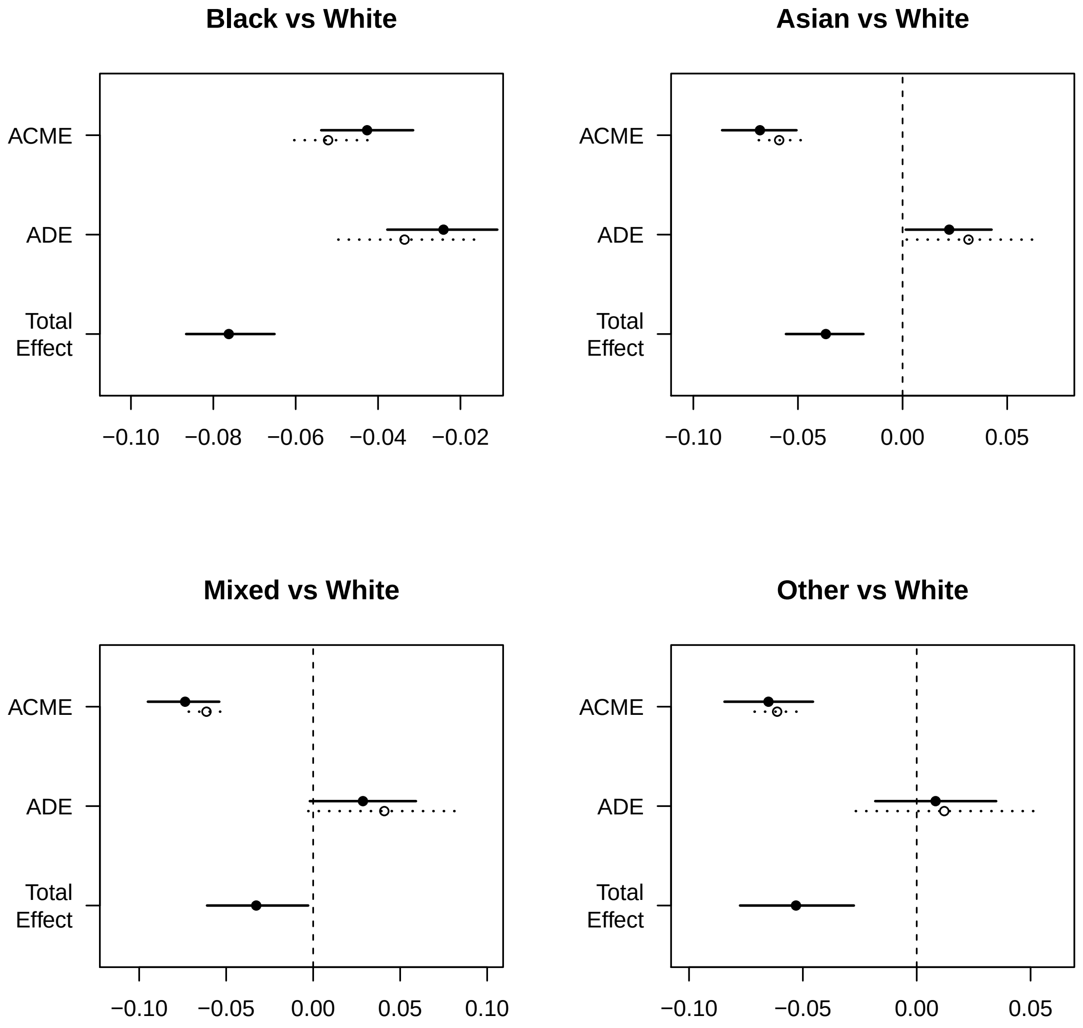

Figure S6: Visualization of mediation analysis results (average causal mediation effects (ACMEs) and average direct effects (ADEs)) for own-group ethnic density as a mediator in examining associations between ethnicity and recorded clozapine prescribing, controlling for all covariates in Model 5 of Table S9. The dashed line represents the control group, namely the White group, and solid line represents a treated group, namely an ethnic minority group as stated in the title of each plot.
